# Skin-derived α-synuclein strains from PD, DLB, and MSA induce distinct intracellular pathology and neurodegeneration

**DOI:** 10.1016/j.jbc.2025.111005

**Published:** 2025-12-08

**Authors:** Anupam Raina, Wen Wang, Jose Carlos Gonzalez, Xiaohui Yan, Linda Overstreet-Wadiche, Jacques I. Wadiche, Chun-Li Zhang, Shu G. Chen

**Affiliations:** 1 Department of Pathology, University of Alabama at Birmingham, Birmingham, Alabama, USA; 2 Department of Neurobiology, University of Alabama at Birmingham, Birmingham, Alabama, USA; 3 Department of Molecular Biology, University of Texas Southwestern Medical Center, Dallas, Texas, USA

**Keywords:** alpha-synuclein, biosensor cells, strains, RT-QuIC, neurodegenerative diseases, skin

## Abstract

α-Synuclein (αSyn) aggregates (“strains”) can be detected by seed amplification assays such as real-time quaking-induced conversion from the skin of patients with synucleinopathies including Parkinson’s disease (PD), dementia with Lewy bodies (DLB), and multiple system atrophy (MSA). However, whether skin-derived αSyn strains induce disease-specific pathology in a biological system is unknown. We have identified a human glioblastoma cell line U251 that readily forms intracellular αSyn inclusions upon seeding by exogenous αSyn seeds. These intracellular αSyn inclusions are detergent-insoluble and colocalize with phosphorylated-αSyn at serine 129 (p-αSyn), the pathological hallmark of synucleinopathies. We have engineered a Förster resonance energy transfer–based αSyn biosensor in U251 cells to characterize intracellular aggregation of αSyn and morphology of p-αSyn inclusions seeded by real-time quaking-induced conversion–amplified patient skin αSyn strains. The skin-derived αSyn strains from PD, DLB, and MSA patients are capable of inducing intracellular αSyn aggregation characterized by Förster resonance energy transfer–positive inclusions colocalized with p-αSyn. Interestingly, PD skin-amplified strains are more bioactive, which induce a greater pathological burden and a distinct p-αSyn inclusion morphology from DLB skin-amplified strains. Furthermore, the skin-amplified αSyn strains induce neuronal inclusions and trigger degeneration of induced neurons reprogrammed from U251 biosensor cells. Finally, biosensor cell-propagated PD skin αSyn strains induce higher *in vitro* seeding activity than DLB skin αSyn strains, indicating a strain-specific relationship between intracellular pathological αSyn burden and *in vitro* seeding activity. In conclusion, αSyn strains derived from PD, DLB, and MSA patient skin are bioactive, pathologically distinct, and trigger neurodegeneration. Our findings emphasize the importance of studying tissue- and strain-specific pathogenesis of synucleinopathies.

The accumulation of misfolded and aggregated alpha-synuclein (αSyn) in the brain is a pathological hallmark of a group of neurodegenerative diseases collectively called synucleinopathies, which include Parkinson’s disease (PD), dementia with Lewy bodies (DLB), and multiple system atrophy (MSA) (1, 2, 3, 4, 5). Aggregated αSyn is invariably present in neuronal inclusions known as Lewy bodies and Lewy neurites in PD and DLB (1) and in glial cytoplasmic inclusions known as Papp-Lantos bodies in MSA (2, 3, 4). Lewy bodies and Lewy neurites are round, oval, or irregularly shaped filamentous inclusions present in the cytosol of neurons in the substantia nigra, cingulate cortex, and other brain regions (1). In MSA, glial cytoplasmic inclusions are primarily found in the cytosol of oligodendrocytes, whereas filamentous inclusions are occasionally detected in neurons with round, oval, C-shape, or ring-like inclusions in the frontal cortex, dentate fascia, substantia nigra, or pontine nucleus (2, 3, 4). More than 90% of aggregated αSyn in these pathological inclusions is phosphorylated at serine 129 (p-αSyn) in a diseased brain, whereas in a healthy brain only about 4% of αSyn is phosphorylated at this site (6, 7). Studying cellular mechanisms underlying the formation of distinct p-αSyn-containing intracellular inclusions may further our understanding of pathogenesis of individual synucleinopathies.

αSyn is an intrinsically disordered protein (8) that can adopt different conformations (9, 10). It is enriched and localized in the presynaptic terminals of neurons and is presumably involved in the recycling of synaptic vesicles and synaptic transmission (11, 12). In a healthy intracellular environment, soluble αSyn exists as a natively unfolded monomer (13). However, in a pathological intracellular environment, monomeric soluble αSyn tends to aggregate into toxic cross-ß-sheet-rich oligomers and assemblies (14, 15, 16, 17, 18) that is a characteristic of an amyloid (19, 20).

Aggregated αSyn assemblies have been shown to behave as a strain with prion-like properties, which was first shown to propagate from brain into grafted neurons in PD patients (21, 22), followed by neuron-to-neuron or cell-to-cell (23, 24) propagation, and transmission between different brain regions *in vivo* (25, 26). Exogenous αSyn strains can also seed the aggregation of intracellular αSyn into Lewy body-like inclusions in cultured cells and neurons (26, 27, 28, 29, 30, 31).

In prior studies, *de novo* generated cross-ß-sheet-rich fibrils using recombinant αSyn (32, 33) (commonly known as recombinant preformed fibrils or rPFF) have been used by investigators to study PD pathogenesis in cultured cells and neurons (27, 28). rPFF can exist as polymorphic assemblies that possess different toxicity and seeding activity *in vitro*, in cells, in neurons, and *in vivo* (34, 35, 36). These studies led to the hypothesis that distinct strains of αSyn might underlie the pathological and clinical heterogeneity observed in synucleinopathies. Consequently, rPFF, patient brain homogenate or αSyn assemblies amplified from patient brain homogenate by protein misfolding cyclic amplification assay (PMCA) (another seed amplification platform) were used to study disease-specific pathology, biochemical characterization of conformers by partial digestion with proteinase-K, seeding activity *in vitro*, *in vivo*, and in cells or neurons (37, 38, 39, 40, 41, 42). These studies in combination with cryo-electron microscopy studies revealed that distinct pathologies were triggered by brain homogenates and brain-amplified αSyn strains in comparison to rPFF (43, 44, 45), suggesting the importance of using patient-derived αSyn strains for studying disease pathology.

We have previously reported that pathological αSyn aggregates in PD, DLB, and MSA patient skin can be robustly detected by real-time quaking-induced conversion (RT-QuIC) assay of patient skin homogenates *in vitro* (46, 47, 48). However, disease-specific pathological features triggered by skin αSyn strains in a biological system remain unknown.

In this study, we first aimed to generate a human brain-derived cell model that closely mimics αSyn inclusion pathology under physiological conditions. We screened different human glioblastoma cell lines that rapidly forms detergent-insoluble intracellular p-αSyn inclusions upon seeding by exogenous rPFF and generated a Förster resonance energy transfer (FRET)–based stable monoclonal αSyn biosensor U251 glioblastoma cell line, which was also amenable to reprogramming toward functional neurons by Neuronal Differentiation 1 (NEUROD1) transcription factor. Then, we investigated the biological and pathological features of skin-amplified and brain-amplified αSyn strains from PD, DLB, and MSA patients in U251 biosensor cells and U251 biosensor cell-derived neurons using FRET-Flow cytometry, high-content confocal imaging, and RT-QuIC assay. The morphology of intracellular p-αSyn inclusions was meticulously classified in U251 biosensor cells. We report that PD patient skin-amplified αSyn strains trigger higher intracellular pathological burden of p-αSyn inclusion and distinct morphology in comparison to the DLB patient skin-amplified and PD patient brain-amplified αSyn strains. Inclusion morphology of DLB and MSA patient skin-amplified αSyn strains mimic those of DLB and MSA patient brain-amplified αSyn strains. We also find that patient skin-amplified αSyn strains are capable of inducing neuronal inclusions and cause degeneration of U251 reprogrammed neurons. Following propagation of patient skin-derived αSyn strains in U251 biosensor cells and subsequent isolation of intracellular αSyn aggregates, we find that biosensor cell-propagated PD skin αSyn strains induce higher seeding activity *in vitro* than DLB skin and PD brain αSyn strains, suggesting a direct relationship between strain-specific intracellular pathological burden and its seeding activity *in vitro*.

## Results

### Engineering FRET-based αSyn biosensor cell line to detect exogenous αSyn strains

**Figure 1:**
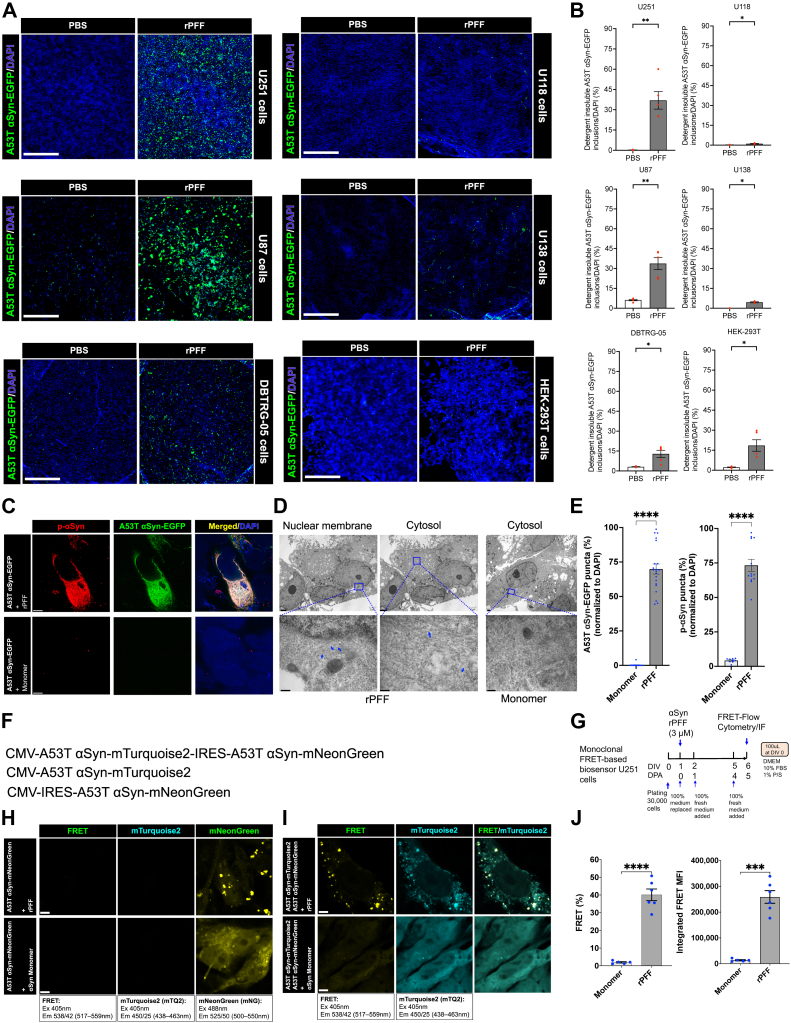
**Rationally designed FRET-based αSyn biosensor U251 cells detect intracellular αSyn aggregation and pathological p-αSyn inclusions**. *A*, representative images showing screening of the indicated cell lines for detergent-insoluble A53T αSyn-EGFP inclusions after seeding with αSyn rPFF. A53T αSyn-EGFP aggregates are shown in *green* (puncta), whereas nuclei are shown in *blue*. Cells were analyzed at day *in vitro* (DIV) 4, 3 days postapplication (DPA) of rPFF. Scale bars: 1 mm. *B*, quantification of detergent-insoluble A53T αSyn-EGFP inclusions. Each dot corresponds to a replicate (well) where on an average 6000 to 21,000 cells were analyzed; N = 4 to 5 per group from two independent experiments. *C*, representative confocal images showing pathological detergent-insoluble A53T αSyn aggregates (*green*) that are phosphorylated (*red*). Cells were analyzed after 5 DIV, 4 DPA of rPFF. Scale bars: 5 μm. *D*, representative TEM images of cellular sections showing fibrillar-like assemblies (*blue arrows*) around nuclear membrane and in the cytosol of cells expressing A53T αSyn-EGFP treated with rPFF and the absence of fibrillar-like assemblies in αSyn monomer treated cells. Scale bars: 2 μm for 3,200x and 0.2 μm for 500,00x magnified images. *E*, quantification of cells with detergent-insoluble A53T αSyn-EGFP puncta (*left*) and p-αSyn positive puncta (*right*) at DPA 4. Each dot corresponds to a replicate where 15,000 to 25,000 cells were analyzed; N = 3 to 4 per group from four independent experiments. *F*, the lentiviral vectors for the expression of A53T αSyn-mTurquoise 2 and A53T αSyn-mNeonGreen (FRET pair) or A53T αSyn-mTurquoise 2 (FRET donor alone) or A53T αSyn-mNeonGreen (FRET acceptor alone) in U251 cells. *G*, experimental design showing the timelines of αSyn-rPFF application on monoclonal U251 αSyn biosensor cell line and using FRET-flow cytometry to select a cell clone that show the highest FRET-based bioactivity. *H* and *I*, representative confocal images showing no bleedthrough of A53T αSyn-mNeonGreen into the FRET channel and rPFF-induced FRET in biosensor cells. Scale bars: 5 μm. *J*, quantification of percent FRET-positive cells and integrated FRET median fluorescence intensity (MFI) in αSyn rPFF and monomer treated biosensor cells by FRET-flow cytometry. Each dot corresponds to a replicate where 22,000 to 25,000 FRET biosensor single cells were analyzed; N = 6 per group from two independent experiments. Statistical analysis was performed by unpaired two-tailed Mann–Whitney test or unpaired two-tailed student’s *t* test, and Welch’s correction was applied when variances were found to be significant. Data are presented as mean ± SEM. A *p*-value < 0.05 was considered significant. Significant differences are indicated by ∗*p* < 0.05, ∗∗*p* < 0.01, ∗∗∗*p* < 0.001, and ∗∗∗∗*p* < 0.0001. Statistical power is > 0.90 between groups with *p*-value < 0.05. FRET, Förster resonance energy transfer; rPFF, recombinant preformed fibrils; αSyn, alpha-synuclein; p-αSyn, phosphorylated αSyn at serine 129.

To study patient-skin derived αSyn strains in a biological system, we engineered a FRET-based αSyn biosensor cell line that detected intracellular αSyn aggregation. To identify a cell line that robustly aggregates intracellular αSyn by seeding with exogenous αSyn proteopathic seeds and is easily maintained in culture, we tested different glioblastoma cell lines because they are derived from human brain and have negligible levels of endogenous αSyn (49) which is known to interfere with αSyn seeding and aggregation in engineered cells (50). Human αSyn carrying the disease-causing A53T mutation is linked to early-onset PD (51) and has been reported to accelerate αSyn oligomerization *in vitro* in comparison with A30P and wildtype (WT) αSyn that eventually form Lewy body-like fibrils at higher concentrations (15, 33, 52, 53). Therefore, we chose human A53T αSyn and tagged it with EGFP (A53T αSyn-EGFP) to be constitutively expressed in human glioblastoma cell lines U251, U118, U87, U138, DBTRG-05, and a human embryonic kidney (HEK) 293T cell line using lentivirus-mediated transduction. Freshly sonicated recombinant αSyn rPFF, commonly used as proteopathic αSyn seeds (27, 28) was applied to the cell culture medium at 1 μM. Treatment of cells with PBS was used as a control. Three days postapplication (DPA) of rPFF seeds, soluble proteins were removed from the cells by *in situ* extraction with detergent resulting in bright detergent-insoluble intracellular A53T αSyn-EGFP positive inclusions visualized by fluorescence microscopy (Fig. S1). A53T αSyn-EGFP inclusions were normalized to the total number of cells (Fig. S2), and percentage of detergent-insoluble αSyn inclusions were reported. Among all cell lines tested, U251 cell line showed the highest percentage of detergent-insoluble αSyn inclusions induced by exogenous rPFF seeds followed by U87, HEK-293T, DBTRG-05, U138, and U118 cell line (Fig. 1, *A* and *B*). Furthermore, U251 cell line reported highest number of cells survived after 3 DPA of rPFF, followed by HEK-293T, U118, DBTRG-05, U138, and U87 (Fig. S2). rPFF-treated cells showed similar cell survival as compared to the PBS-treated cells (Fig. S2). Overall, our results suggest that human U251 glioblastoma immortalized cell line provides an ideal cellular milieu for intracellular αSyn aggregation upon seeding by rPFF under quasi-physiological conditions, and it allows rapid *in situ* detection of detergent-insoluble intracellular αSyn inclusions.

Next, monoclonal U251 cell lines were generated that constitutively express either A53T αSyn-EGFP or WT αSyn-EGFP. Cell culture conditions were optimized for enhancing the number of detergent-insoluble αSyn inclusions in U251 cells upon seeding with rPFF (Fig. S3). Serum concentration and instrument-specific sonication parameters were observed to affect rPFF seeding efficiency in U251 cells (Fig. S3, *C* and *H*). A dose-dependent increase of intracellular αSyn inclusions were observed with increasing concentration of exogenous rPFF in the medium (Fig. S3, *J* and *K*). To examine if the detergent-insoluble inclusions induced by rPFF are biochemically and pathologically similar to those present in diseased brains, immunofluorescence was performed to detect pathological p-αSyn 4 days after rPFF application on U251 cells followed by *in situ* removal of soluble proteins with detergent extraction. Cells treated with αSyn monomer were used as control. p-αSyn was strongly detected in the detergent-insoluble A53T or WT αSyn-EGFP positive inclusions (Figs. 1*C* and S4*A*). Further, TEM revealed fibril-like assemblies around the nuclear membrane and in the cytosol (Fig. 1*D*, blue arrows) of rPFF-treated U251 cells with A53T αSyn inclusions that is consistent with the subcellular localization of p-αSyn inclusions (Fig. 1*C*). Moreover, the width of these fibril-like assemblies in cells ranged from 9.9 to 10.7 nm and remain undetected in the αSyn monomer-treated cells (Fig. 1*D*, monomer, blue box). Phosphorylated αSyn detergent-insoluble inclusions were around 2.3-fold higher in A53T αSyn-EGFP expressing U251 cells (Fig. 1*E*) than the WT αSyn-EGFP expressing cells (Fig. S4*B*). Total number of WT αSyn-EGFP (Fig. S4*B*) or A53T αSyn-EGFP (Fig. S4*C*) expressing cells that survived after 4 days posttreatment with monomer and rPFF were not significantly different. To examine whether U251 cells release pathological αSyn seeds, RT-QuIC assay of the cell culture medium (supernatant) was performed. rPFF or PBS or αSyn monomer was applied to A53T αSyn-EGFP expressing U251 cells, and after several medium changes, supernatant was collected at day 15 and diluted 100,00x in N2/PBS for RT-QuIC assay (Fig. S4*D*). rPFF induced intracellular αSyn inclusions that were quantified (Fig. S4*E*). Cell survival was significantly reduced at day 15 with rPFF treatment (Fig. S4*F*). Seeding activity was strongly detected in the supernatant of rPFF-treated cells reaching peak thioflavin T (ThT) fluorescence within 60 h but not in the supernatant of cells treated with αSyn monomer or PBS (Fig. S4*G*). These data suggest that αSyn-inclusion-containing U251 cells appear to release αSyn aggregates into the culture medium. However, if the released αSyn aggregates are internalized and induce αSyn aggregation in the neighboring cells remains to be investigated. Overall, our results indicate that rPFF seeds induce the formation of pathological p-αSyn-positive inclusions in the cytosol and around the nuclear membrane in U251 cells, with subsequent release of proteopathic αSyn aggregates.

Finally, to enhance the detection sensitivity of intracellular αSyn aggregation in response to exogenous proteopathic αSyn seeds, FRET-based αSyn biosensor U251 cells were generated. We constructed a bicistronic lentiviral vector containing both FRET donor and acceptor transgenes fused to the C terminus of A53T αSyn (Fig. 1*F*). CMV promoter controlled the expression of A53T αSyn-mTurquoise2 (αSyn-mTQ2/αSyn-CFP) and IRES controlled the expression of A53T αSyn-mNeonGreen (αSyn-mNG/αSyn-YFP). Monocistronic lentiviral vectors containing either αSyn-mTQ2 or αSyn-mNG were used as negative controls (Fig. 1*F*). αSyn biosensor U251 cell clone was generated, and 5 days after application of rPFFs, FRET-Flow cytometry was performed (Fig. 1*G*). Monomeric αSyn and rPFF seeds were used as negative and positive controls, respectively. Strategically, a subset of biosensor cells expressing αSyn-mNG was selected from single-cell population (Fig. S5*A*). We found that the control cell line groups expressed either αSyn-mTQ2 or αSyn-mNG, but biosensor cells expressed both (Fig. S5*B*). Control and biosensor cells treated with αSyn monomer were excited to detect bleed-through of either αSyn-mNG or αSyn-mTQ2 into FRET channel using which FRET gates were drawn (Fig. S5, *C* and *D*). Upon confocal imaging of cells that expressed only acceptor (αSyn-mNG), there was no bleed-through of mNG into FRET channel (Fig. 1*H*) confirming the specificity of FRET-flow cytometry. When rPFF was applied on biosensor cells, αSyn aggregates were detected in FRET channel that were distinct from the background fluorescence of cells treated with αS monomer (Fig. 1*I*). Using FRET-flow cytometry, we found that 3 μM rPFF induced FRET in 40.1% of biosensor cells with 19-fold higher integrated FRET median fluorescence intensity (MFI) than the cells treated with αSyn monomer (Fig. 1*J*). Taken together, we successfully generated U251 αSyn biosensor cell line that efficiently detects exogenous αSyn proteopathic seeds.

### Patient skin αSyn strains are amplified into polymorphic amyloid fibrils by RT-QuIC

**Figure 2:**
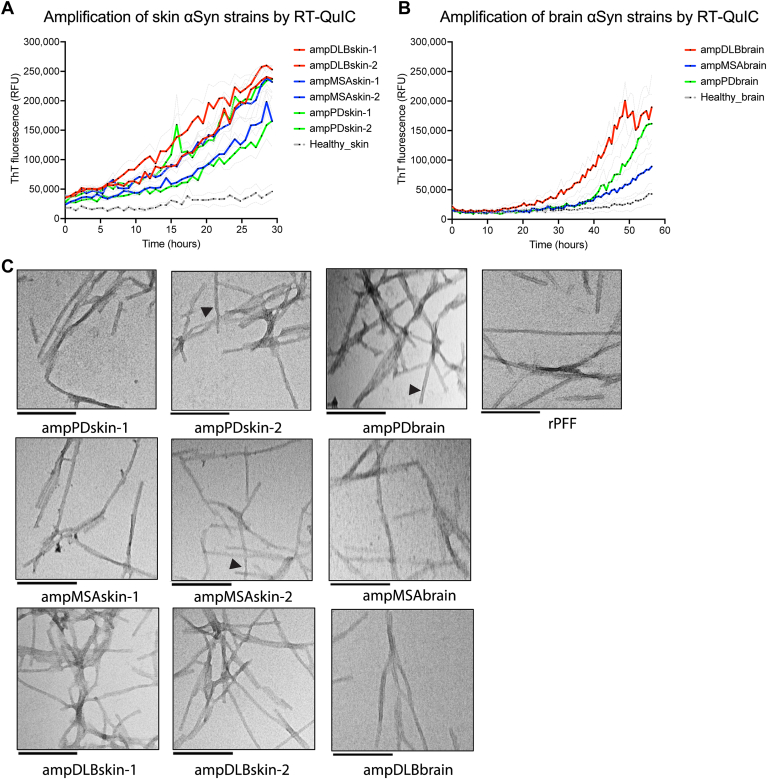
**Amplification of patient skin- and brain-derived PD, DLB, and MSA αSyn strains by RT-QuIC**. *A* and *B*, amplification of αSyn aggregates from PD, DLB, and MSA patient skin and brain homogenates using RT-QuIC. Data are presented as mean ± SEM (*gray line*), and each dot represents data analyzed from N = 3 replicates (wells). *C*, representative TEM images of PD, DLB, and MSA patient αSyn strains amplified from skin or brain homogenates. *Black arrowheads* indicate twists. Scale bars: 200 nm. DLB, dementia with Lewy bodies; αSyn, alpha-synuclein; MSA, multiple system atrophy; PD, Parkinson’s disease; RT-QuIC, real-time quaking-induced conversion; ThT, thioflavin T.

The structure of patient skin-derived αSyn fibrils has not been previously studied and remains to be resolved. To unravel the ultrastructural morphology of skin αSyn fibrils for investigating the pathology of patient skin αSyn strains in U251 biosensor cells, we generated skin-derived αSyn amyloid fibrils (skin-amplified αSyn strains) from two cases each of PD, DLB, and MSA skin homogenates by RT-QuIC seed amplification assay as previously reported (46, 47, 48). Similarly, one case each of PD, DLB, and MSA patient brain homogenate was used to generate brain-derived αSyn amyloid fibrils (brain-amplified αSyn strains) by RT-QuIC. Healthy skin and brain homogenates were used as controls to monitor the specificity of RT-QuIC assay. RT-QuIC run was completed when ThT fluorescence readout of skin and brain homogenates from healthy subjects reached a baseline set-point of 50,000 relative fluorescence units (RFUs). Patient skin- and brain-derived αSyn strains resulted in increased ThT fluorescence readout ranging from 100,000 to 260,000 RFU within 29 h (Fig. 2*A*) and 56 h (Fig. 2*B*), respectively. These amplified skin and brain αSyn aggregates were subsequently purified by ultracentrifugation at 186,000×*g* to exclude monomeric αSyn. TEM analysis of skin-amplified αSyn aggregates revealed that they consist mostly of fibrillar (straight or twisted) or ribbon-like (flat) structures (Fig. 2*C*) based on the classification of fibrils as previously reported (35). A very small subpopulation of skin- and brain-amplified PD and MSA αSyn fibrils exhibited twists (Fig. 2*C*, black arrowheads), suggesting they are fibrillar-like, while the majority of fibril population appeared to be flat, suggesting they are ribbon-like (Fig. 2*C*). The DLB skin- and brain-amplified αSyn fibrils as well as *de novo* generated rPFF appeared to be mostly straight, suggesting they are fibrillar-like (Fig. 2*C*). Taken together, we report that PD and MSA skin-amplified αSyn fibrils are mostly ribbon-like with a subpopulation of fibrillar-like amyloid fibrils, whereas DLB skin-amplified fibrils are fibrillar-like.

### Patient skin-amplified αSyn strains induce distinct inclusion pathology in U251 biosensor cells

**Figure 3:**
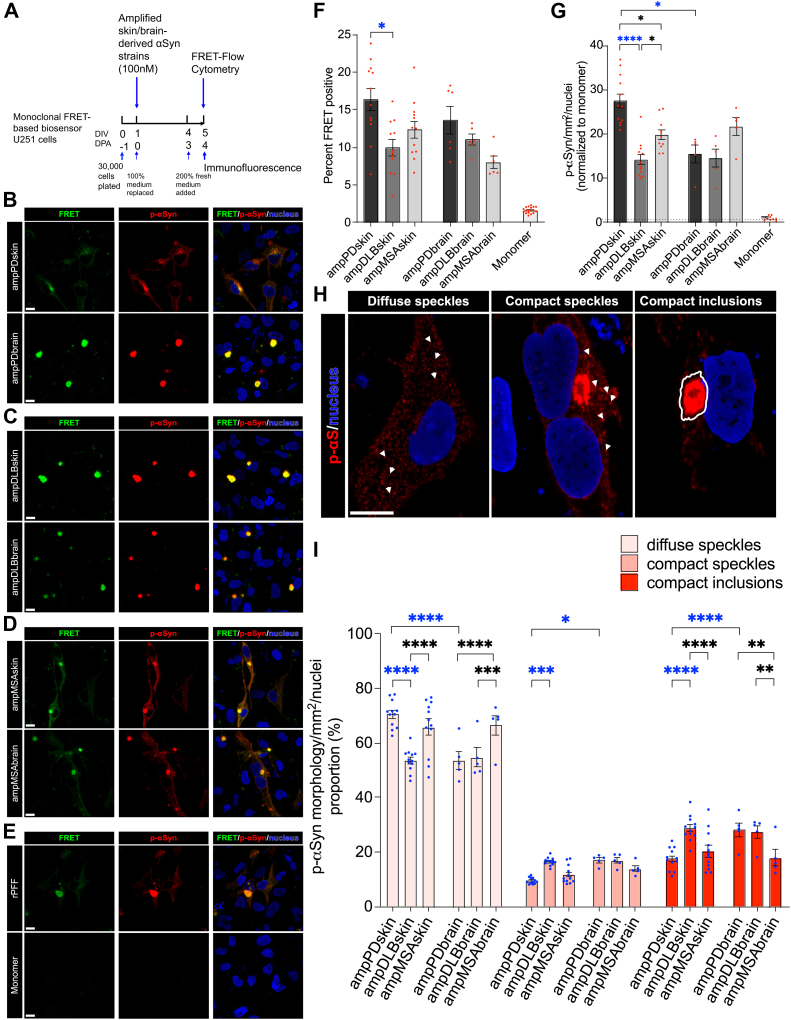
**Patient skin-amplified αSyn strains induce distinct inclusion pathology in biosensor cells**. *A*, experimental design showing the timelines of the application of skin or brain-amplified αSyn strains with lipofectamine on monoclonal FRET-based biosensor U251 cells. *B*–*E*, and *H*, representative confocal images showing large FRET and p-αSyn positive Lewy body-like aggregates in the cytosol mostly tethered to the nuclear membrane and classification (*H*) of p-αSyn aggregate morphology These inclusions were not present in αSyn monomer-treated cells. Scale bars: 10 μm. *F*–*G*, and *I*, quantification of percent FRET-positive cells (*F*), total intracellular p-αSyn aggregate load (*G*), and proportions of p-αSyn aggregate morphology (*I*). Data are presented as mean ± SEM, and each dot corresponds to data analyzed from 20,000 to 25,000 (*F*) FRET-positive cells and 6000 to 10,000 (*G* and *I*) biosensor cells in a well from two to three independent experiments; N = 12 (*F*) and N = 10 to 12 (*G* and *I*) from the skin-amplified strains of two cases, N = 6 (*F*) and N = 5 (*G* and *I*) from the brain-amplified strains of one case, and N = 9 (*G*) from αSyn monomer group. Statistical analysis was performed by ordinary one-way or two-way (I) ANOVA with Tukey’s multiple comparison test or Brown-Forsythe and Welch ANOVA with Dunnet’s T3 multiple comparison test (*F* and *G*). A *p* value < 0.05 was considered significant. Significant differences are indicated by ∗*p* < 0.05, ∗∗*p* < 0.01, ∗∗∗*p* < 0.001, ∗∗∗∗*p* < 0.0001. Statistical power is > 0.90 between groups with *p*-value < 0.05. αSyn, alpha-synuclein; FRET, Förster resonance energy transfer; p-αSyn, phosphorylated αSyn at serine 129.

Using U251 cells expressing A53T αSyn FRET biosensors, we sought to determine if patient skin-amplified PD, DLB, and MSA αSyn strains are pathologically active that can seed intracellular αSyn aggregation in biosensor cells. 100 nM of purified PD, DLB, and MSA skin and brain-amplified αSyn strains were applied to U251 biosensor cells to seed intracellular aggregation of αSyn, along with rPFF and αSyn monomer as respectively positive and negative controls (Fig. 3*A*). Immunofluorescence of pathological p-αSyn inclusions and FRET-flow cytometry was performed at day 4 posttreatments with patient-derived αSyn strains. Strong FRET signals were observed in U251 biosensor cells upon seeding by all patient-derived αSyn strains and rPFF but not the αSyn monomer control, which overlapped with the p-αSyn positive inclusions (Fig. 3, *B*–*E*). When exogenous αSyn strains induce aggregation of intracellular αSyn, biosensor cells generate a FRET signal that is detected and quantified as the percent of biosensor cells positive for FRET (Fig. 3*F*). Remarkably, skin-amplified PD strains were more bioactive, as shown by higher percentage of cells positive for FRET signals, than skin-amplified DLB strains in inducing aggregation of intracellular αSyn (Fig. 3*F*). There was no significant difference in FRET positivity between skin-amplified αSyn strains and brain-amplified αSyn strains from PD, DLB, and MSA patients (Fig. 3*F*). Our results suggest that patient skin-amplified PD, DLB, and MSA αSyn strains are biologically active, and skin-amplified PD strains were more bioactive than skin-amplified DLB strains in U251 biosensor cells.

To further examine morphological features of pathological intracellular αSyn aggregates in biosensor cells seeded by patient skin- and brain-amplified strains, immunofluorescence of p-αSyn inclusions in biosensor cells was performed at day 4 posttreatments with skin and brain-amplified strains and analyzed by high-content confocal microscope. FRET-positive inclusions were completely colocalized with pathological p-αSyn inclusions (Fig. 3, *B*–*E*). Interestingly, skin-amplified PD strains induced significantly more p-αSyn inclusions than brain-amplified PD strains (Fig. 3*G*). Remarkably, skin-amplified PD strains induced significantly higher burden of p-αSyn pathology than skin-amplified DLB and MSA strains (Fig. 3*G*). Moreover, skin-amplified MSA strains also induced more p-αSyn pathology than skin-amplified DLB strains (Fig. 3*G*). No significant differences were observed between skin- and brain-amplified strains in DLB and MSA groups in terms of their ability to induce p-αSyn positive inclusions (Fig. 3*G*). To enhance our understanding of p-αSyn inclusion morphology, we classified intracellular p-αSyn assemblies into four classes of morphologies based on mean fluorescence intensity (MFI) and size of the assembly, namely, diffuse speckles, diffuse inclusions, compact speckles, and compact inclusions (Figs. 3*H* and S6*A*). Lower MFI (≤14,999) was termed as diffuse, whereas higher MFI (≥15,000) was termed as compact. When a p-αSyn positive assembly covers a smaller area (0.5–4.5 μm) in a cell, it was termed as a speckle, whereas when it covers a larger area (4.6–35 μm), it was termed as an inclusion. Using this classification, we found that skin- and brain-amplified αSyn strains induced more diffuse and less compact p-αSyn pathology overall (Fig. 3*I*). Remarkably, we found that skin-amplified PD and MSA strains induced similar p-αSyn morphologies with more diffuse speckles and less compact inclusions than skin-amplified DLB strains (Fig. 3*I*). In contrast, PD and DLB brain-amplified strains induced similar p-αSyn morphologies with more compact inclusions and less diffuse speckles than MSA brain-amplified strains (Fig. 3*I*), fortifying the previously reported αSyn strain-specific differences between PD, DLB, and MSA that used αSyn strains purified by sarkosyl from patient brain homogenate (43, 54) or directly used patient brain homogenate (38, 55). Interestingly, PD skin-amplified strains differed from PD brain-amplified strains, the latter resulted in less diffuse speckles but more compact inclusions (Fig. 3*I*). DLB and MSA skin-amplified αSyn strains induced p-αSyn morphologies similarly as brain-amplified strains in U251 biosensor cells (Fig. 3*I*). The proportion of p-αSyn diffuse inclusions was very small and ranged from 1 to 4% that was not significantly different among groups of skin- and brain-amplified strains (Fig. S6, *A* and *B*). Additionally, there was no significant drop in the cell count after 4 days postapplication with patient skin-amplified strains (Fig. S6*C*). However, MSA brain-amplified strains appeared to reduce cell survival as compared to MSA skin-amplified and PD brain-amplified strains (Fig. S6*C*). Taken together, our results indicate that PD, DLB, and MSA patient-amplified αSyn strains are biologically active and induce distinct pathological p-αSyn inclusion morphology in U251 αSyn biosensor cells. Furthermore, skin-amplified αS strains derived from PD patients can be potentially discriminated from DLB patient skin-amplified αSyn strains by analyzing FRET bioactivity and p-αSyn inclusion morphology in U251 biosensor cells.

### Patient skin-amplified αSyn strains trigger neurodegeneration

**Figure 4:**
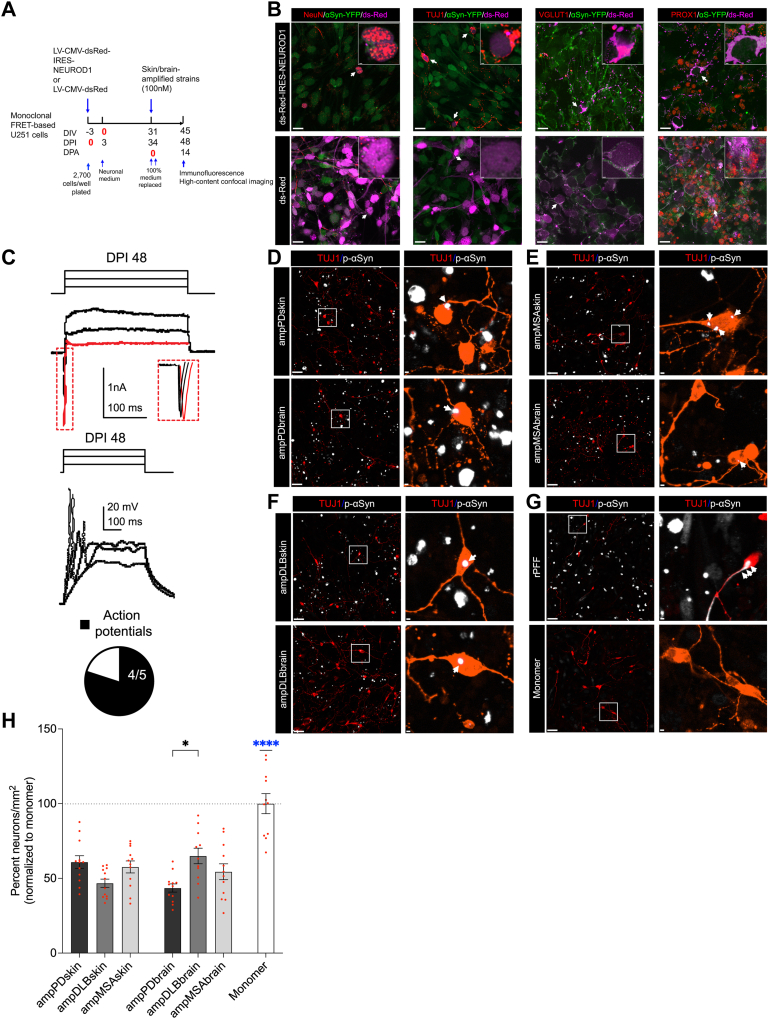
**Patient skin-amplified αSyn strains trigger the formation of intraneuronal p-αSyn inclusions and are neurotoxic**. *A*, experimental design showing the timelines for generation of induced neurons from U251 FRET-based biosensor cells by reprogramming with lentiviral transduction of NEUROD1 and neuronal differentiation followed by application of skin- or brain-amplified αSyn strains with lipofectamine. *B*, representative confocal images showing the expression of TUJ1 and mature neuronal markers NeuN and VGLUT1 but not PROX1 at DPI 48. *White arrows* indicate the expanded inset image showing the subcellular localization of neuronal markers. Scale bars: 20 μm and 1 μm. *C*, a representative voltage clamp recording at −80 mV from a NEUROD1-positive cell at DPI 48 revealing large sodium currents in response to 50, 60, and 70 mV steps (300 ms); scale: 1 nA/100 ms. The sodium current in response to the 50 mV step is expanded and highlighted in *red*. Below, voltage traces from a NEUROD1-positive cell at DPI 48 in response to current injections (10, 30, 50 pA; 500 ms) revealing action potentials; scale 20 mV/100 ms. The pie chart highlights the number of NEUROD1-positive cells with action potentials. *D*–*G*, representative confocal images showing intraneuronal p-αSyn inclusions that were mostly localized in the soma. *White squares* indicate expanded image on the *right* showing the morphology and subcellular localization of p-αSyn inclusions as indicated by *white arrows*. Scale bars: 50 μm and 3 μm. *H*, quantification of surviving neurons at DPA 14. Data are presented as mean ± SEM, and each dot corresponds to data analyzed from 180 to 700 neurons in a well. N = 10 to 12 from the skin-amplified strains of two cases and N = 11 to 12 from the brain-amplified strains of one case, and N = 11 to 12 from monomer group from two independent differentiations. Statistical analysis was performed by ordinary one-way ANOVA with Tukey’s multiple comparison test. A *p-*value < 0.05 was considered significant. Significant differences are indicated by ∗*p* < 0.05 and ∗∗∗∗*p* < 0.0001. Statistical power is > 0.90 between groups with *p*-value < 0.05. αSyn, alpha-synuclein; FRET, Förster resonance energy transfer; NEUROD1, neuronal differentiation 1; p-αSyn, phosphorylated αSyn at serine 129; DPI, days postinfection; DPA, days postapplication.

We have shown that patient skin-amplified αSyn strains are biologically active and mimic p-αSyn inclusion pathology of PD, DLB, and MSA in A53T αSyn expressing U251 biosensor cells. However, it is not known if such p-αSyn inclusion pathology is also recapitulated in human neurons. Therefore, we sought to reprogram human U251 glioblastoma biosensor cells into functional neurons while preserving the sensitive detection of αSyn aggregation. NEUROD1 has been reported to reprogram cultured human astrocytes in an Alzheimer’s disease mouse model into functional glutamatergic neurons (56). Furthermore, U251 cells could be reprogrammed into functional glutamatergic neurons simply by NEUROD1 ectopic expression (57, 58). Here, we used lentiviral vector to overexpress NEUROD1 in U251 glioblastoma biosensor cells using dsRed as a reporter (Fig. 4*A*). Indeed, NEUROD1 expressing U251 cells developed neuronal morphology with long neurites and were positive for neuronal marker TUJ1 and mature neuron marker NeuN at 48 days postinfection (DPI) (Fig. 4*B*). Moreover, these mature neurons were VGLUT1 positive (Fig. 4*B*) indicating that they are glutamatergic neurons. Surprisingly, U251 biosensor cells endogenously express PROX1 (Fig. 4*B*), which is a granule cell nuclear marker. Remarkably, PROX1 was not detected in dsRed positive (NEUROD1 expressing) reprogrammed neurons (Fig. 4*B*, upper panel inset). A majority of U251 biosensor cells that remain positive for PROX1 were either untransduced (Fig. 4*B*, upper panel, dsRed negative) or do not express NEUROD1 (Fig. 4*B*, lower panel, control), suggesting that NEUROD1-mediated reprogramming results in the loss of PROX1 phenotype in reprogrammed neurons. Patch-clamp generated action potentials in 80% of NEUROD1-positive cells as high sodium currents were observed on DPI 48 (Fig. 4*C*), suggesting successful reprogramming of U251 biosensor cells into human-induced biosensor neurons (hiBNs). Skin- and brain-amplified αSyn strains were applied to hiBNs on DPI 34 that resulted in the formation of intracellular pathological p-αSyn aggregates in TUJ1 positive (TUJ1+) neurons at DPI 48, which were mostly localized to the soma (cell body and the nucleus) of reprogrammed neurons (Fig. 4, *D*–*G*). Skin- and brain-amplified αSyn strains resulted in more dystrophic bulbous TUJ1+ neurites within 2 weeks in comparison to αSyn monomer control (Fig. 4, *D*–*G*), indicating ongoing neurodegeneration of hiBNs induced by patient skin- and brain-amplified αSyn strains. To assess neurotoxicity, number of surviving neurons were quantified by counting TUJ1+ soma at DPI 48, 2 weeks postapplication of patient-amplified αSyn strains. We found that skin- and brain-amplified strains were neurotoxic to 34 to 54% of total hiBNs as compared to the αSyn monomer-treated neurons (Fig. 4*H*). Brain-amplified PD strains were significantly more neurotoxic than the brain-amplified DLB strains (Fig. 4*H*). In summary, these data show that patient skin- and brain-amplified αSyn strains can trigger the formation of pathological p-αSyn aggregates and neurodegeneration in human U251 biosensor cell-derived neurons.

### Distinct p-αSyn signatures of patient skin αSyn strains dictate seeding activity in vitro

**Figure 5:**
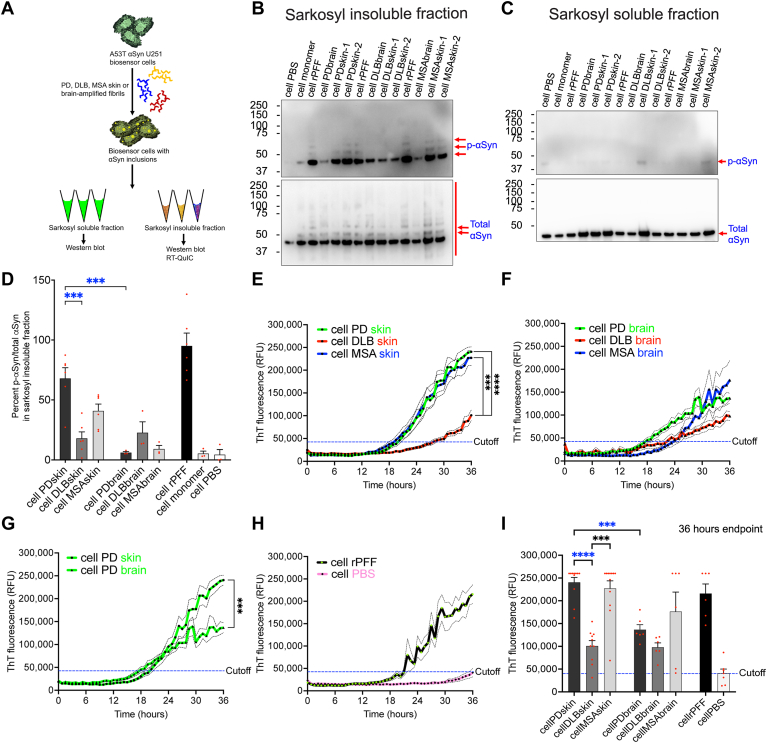
**Intracellular p-αSyn signatures induced by patient skin αSyn strains dictate their seeding activity *in vitro.****A*, schematic of the generation and purification of U251 biosensor cell-derived patient skin or brain αSyn strains. *B* and *C*, Western blots showing pathological p-αSyn and total αSyn detected in sarkosyl detergent-insoluble (*B*) and sarkosyl detergent-soluble fraction (*C*) from U251 biosensor cells seeded with patient skin- or brain-amplified αSyn strains. *D*, quantification of percent p-αSyn normalized to total αSyn in sarkosyl detergent-insoluble fraction from Western blots by densitometric analysis. Data are presented as mean ± SEM, and each dot corresponds to data analyzed using p-αSyn and total αSyn protein band densities in that sample group. N = 6 replicates from biosensor cell-derived skin αSyn strains of 2 cases, N = 3 replicates from biosensor cell-derived brain αSyn strains of 1 case, N = 3 replicates each from biosensor cell-derived αSyn monomer and D-PBS, and N = 6 replicates from biosensor cell-derived rPFF. *E*–*I*, RT-QuIC assay (*E–H*) of biosensor cell-derived patient skin or brain αSyn strains showing their *in vitro* seeding activity. Cutoff was set just below 50,000 RFU using biosensor cell-derived fraction that was initially treated with D-PBS. Quantification of endpoint ThT fluorescence (*I*) at 36-h time-point from RT-QuIC assay showing *in vitro* seeding activity of patient skin- and brain-amplified αSyn strains following propagation in U251 biosensor cells. Data are presented as mean ± SEM (*dashed lines* or bars). Each dot corresponds to data analyzed from N = 10 to 12 replicates from biosensor cell-derived skin αSyn strains of two cases, N = 6 replicates from biosensor cell-derived brain αSyn strains of one case, N = 5, and N = 7 replicates for biosensor cell-derived αSyn monomer and D-PBS, respectively, and N = 7 replicates for RT-QuIC internal control with D-PBS quantified at every 45 min interval for 36 h from two independent experiments. Statistical analysis was performed by ordinary one-way ANOVA with Tukey’s multiple comparison test (*D*) or ordinary two-way ANOVA with Tukey’s multiple comparison test (*E–H*) or Brown–Forsythe and Welch ANOVA with Dunnet’s T3 multiple comparison test (*I*). A *p*-value < 0.05 was considered significant. Significant differences are indicated by ∗∗∗*p* < 0.001 and ∗∗∗∗*p* < 0.0001. Statistical power is > 0.90 between groups with *p*-value < 0.05. RT-QuIC, real-time quaking-induced conversion; αSyn, alpha-synuclein; p-αSyn, phosphorylated αSyn at serine 129. ThT, thioflavin T; D-PBS, Dulbecco’s phosphate-buffered saline.

We have shown that patient skin-amplified αSyn strains can seed intracellular aggregation of αSyn into pathological Lewy body-like p-αSyn inclusions in biosensor cells (Fig. 3) and induced biosensor neurons (Fig. 4) that are reminiscent of Lewy body pathology found in PD, DLB, and MSA patient brains. However, it remains unknown if these newly formed intracellular αSyn inclusions generated in biosensor cells have intrinsic seeding activity to further propagate αSyn aggregation. To address this question, biosensor cells were treated with patient skin- and brain-amplified αSyn strains to seed intracellular aggregation of αSyn. When FRET-positive αSyn inclusions were detected at day 4 postapplication of patient-amplified αSyn strains, biosensor cells were lysed and sequentially purified by detergent extraction with NP-40 and sarkosyl (Fig. 5*A*). The detergent-insoluble fraction was then subjected to RT-QuIC assay and immunoblot. On immunoblots, p-αSyn levels were enriched in the detergent-insoluble fraction (Figs. 5*B* and S7*A*) but not in the detergent-soluble fraction (Figs. 5*C* and S7*B*). PD skin αSyn strains triggered higher levels of detergent-insoluble p-αSyn that were distinct from DLB skin and PD brain αSyn strains (Fig. 5, *B* and *D*). On RT-QuIC assay, detergent-insoluble fraction of PBS-treated biosensor cells (cell PBS) was found to be an ideal control as this group showed very minimal increase in ThT fluorescence that was set as a cutoff and endpoint for RT-QuIC reactions (Fig. 5, *D*–*G*). RT-QuIC assay detected significantly higher ThT fluorescence (hence higher *in vitro* seeding activity) from skin and brain αSyn strains derived from biosensor cells in comparison to control after 36 h of RT-QuIC reactions (Fig. 5, *E*–*H*). Comparison of *in vitro* RT-QuIC seeding activity at the endpoint 36th hour revealed significant differences between biosensor cell-amplified PD and MSA *versus* DLB skin αSyn strains. PD skin strains, as well as MSA skin strains exhibited higher seeding activity than DLB skin and PD brain strains (Fig. 5, *E*, *G*, and *I*), which very well partly corresponds to the immunoblot showing higher p-αSyn burden triggered by PD skin αSyn strains than DLB skin and PD brain strains in U251 biosensor cells (Fig. 5*D*). Taken together, we showed that A53T αSyn U251 biosensor cells preserve bioactivity of PD, DLB, and MSA skin and brain αSyn strains which are faithfully propagated *in vitro* by RT-QuIC assay. The p-αSyn pathological burden triggered in U251 biosensor cells by patient skin-amplified αSyn strains dictate seeding activity of biosensor cell-derived patient skin αSyn strains, allowing potential discrimination between PD and DLB strains and establishing a direct relationship between p-αSyn inclusion pathology and seeding activity.

## Discussion

It remains unknown how distinct αSyn strains are generated and propagated that give rise to distinct α-synucleinopathies such as PD, DLB, and MSA. We previously showed that pathological αSyn aggregates are present in PD, DLB, and MSA patient skin, and seeding activity of skin αSyn strains can be robustly detected by RT-QuIC assay *in vitro* (46, 47, 48). However, disease-specific pathological features induced by skin αSyn strains of PD, DLB, and MSA in a biological system have not yet been studied.

In this study, we report the sensitive detection of pathologically distinct αSyn strains derived from PD, DLB, and MSA patient skin that induce unique pathological phenotypes in U251 FRET-based αSyn biosensor cells. Moreover, patient skin αSyn strains trigger degeneration of induced biosensor neurons. Finally, we showed that the intracellular pathological skin-αSyn burden in U251 biosensor cells dictates *in vitro* αSyn seeding activity with a potential to discriminate PD from DLB αSyn strains and thereby establishing a strain-specific relationship between inclusion pathology and seeding activity.

### U251 FRET-based αSyn biosensor cells for disease modeling

Many immortalized cell lines have been used to study αSyn aggregation (59, 60). Differentiation of SH-SY5Y human neuroblastoma cells to neuron-like cells have been used, but they are difficult to grow and maintain and widely differ in neuronal differentiation and functionality when compared across different laboratories, making them less reliable for αSyn aggregation studies (61). By contrast, HEK293T cells have been widely used to study αSyn seeding by exogenous proteopathic seeds, inclusion morphology, and propagation (62). Given that HEK293T cells were isolated from the kidney of a human embryo, the αSyn inclusions in HEK293T cells induced by distinct αSyn strains might not be a good representation of the inclusions originally formed in neurons, astrocytes, or oligodendrocytes in the human brain because it has been shown that intracellular milieu generates distinct pathological strains of αSyn (63). Therefore, we used human brain–derived glioblastoma cell lines that grow rapidly and are easy to maintain. As prior work showed that exogenous αSyn rPFF can seed intracellular aggregation of αSyn to form inclusions (27, 28, 64), we expressed A53T αSyn-EGFP in different glioblastoma cell lines and exposed them to exogenous αS rPFF. Upon screening, we found that human U251 cells can efficiently seed intracellular A53T αSyn-EGFP into large Lewy body-like detergent-insoluble inclusions. Currently, it remains unknown how αSyn rPFF is internalized into U251 cells. We speculate that receptor-mediated endocytosis may be one of the mechanisms underlying the uptake of αSyn fibrils because intracellular αSyn inclusion formation almost completely saturates at 4 μM of exogenous αSyn fibrils (Fig. S3*J*), suggesting the saturation of cell-surface receptors. Heparan sulfate proteoglycans (62) have been claimed to be involved in the internalization of αSyn fibrils in cell lines, as well as LAG3 (65) that remains disputed (66), neuronal LRP1 (67), GPNMB (68), and FAM171A2 (69). However, an extensive and careful study is crucial to precisely identify potential cell-surface receptors that strongly bind and internalize αSyn fibrils into U251 cells.

We subsequently generated monoclonal U251 cells stably expressing A53T or WT αSyn-EGFP. When these cells were exposed to exogenous rPFF, intracellular detergent-insoluble p-αSyn inclusions were readily detected, suggesting efficient seeding by rPFF and formation of pathological A53T or WT p-αSyn inclusions in U251 cells. To our knowledge, rapid *in situ* detection of intracellular detergent-insoluble αSyn inclusion in cells has not been shown by previous studies with existing cellular models of αSyn aggregation. Strikingly, we found that U251 cells allowed rapid detection of bright EGFP-positive intracellular αSyn inclusions following a simple treatment with a nonionic detergent, suggesting that U251 cells can be used for powerful analyses of detergent-insoluble αSyn aggregates inside cells with tools like high-content imaging. To enhance the detection sensitivity of αSyn inclusion formation, a FRET-based biosensor cell model using U251 cells was generated. To express equal proportion of donor and acceptor fluorophores, a bicistronic lentiviral vector was designed that allowed the coexpression of fusion proteins A53T αSyn-mTurquoise2 (αSyn-mTQ2 or αSyn-CFP) controlled by CMV promoter and A53T αSyn-mNeonGreen (αSyn-mNG or αSyn-YFP) controlled by IRES. Using FRET-flow cytometry, single-cell population of U251 biosensor cells was gated to report percent of cells that were FRET positive. We did not detect any bleed through of αSyn-mNG acceptor into FRET channel, neither by FRET-flow cytometry nor by confocal imaging in U251 biosensor cells, confirming an ideal choice of donor and acceptor fluorophores. Intracellular αSyn inclusions in biosensor cells were efficiently detected by FRET-flow cytometry or confocal imaging when rPFF seeds were applied to U251 biosensor cells. This newly generated monoclonal FRET-based αSyn U251 cell model provides a robust platform to investigate intracellular αSyn inclusion pathology induced by patient skin-derived αSyn strains.

### PD, DLB, and MSA skin αSyn strains: Inclusion pathology to seeding relationship

Here, we showed that skin-derived αS strains from PD, DLB, and MSA patients impart distinct bioactivity and inclusion pathology in human U251 biosensor cells. When patient skin αSyn strains were amplified by RT-QuIC, we observed a mixture of mostly straight and a small subset of twisted filaments in PD and MSA skin- and brain-amplified fibrillary structures using TEM which is also reported in prior work in the context of brain-derived αSyn fibrils (3, 40, 42, 54, 70). Mostly, straight filaments were observed in DLB skin- and brain-amplified assemblies. Perren *et al.* showed that PMCA-amplified PD and MSA αSyn strains shared similar profile of partial proteinase-K digestion resembling ribbon-like morphology, which was different than that of PMCA-amplified DLB αSyn strains resembling fibril-like morphology (40) consistent with our findings in the context of TEM imaging. Nevertheless, some studies claim that αSyn strains amplified from brain homogenates are structurally different from αSyn strains present in brain homogenates (70, 71). Notably, these studies have used different salt concentrations or different buffer and at different pH and temperatures to amplify αS strains that has been shown to affect *de novo* generated αSyn fibril conformation (35), which might possibly be one of the reasons for structural differences between these preparations. On the other hand, isolation and purification of αSyn fibrils from patient brain might potentially induce conformational changes in patient αSyn strains (72). Therefore, studies involving patient-derived αSyn assemblies require careful investigation, and the availability of robust biosensor cells may permit discrimination of different αSyn strains under quasi-physiological conditions.

Interestingly, we found that skin-amplified PD strains have a higher FRET-based bioactivity in triggering intracellular αSyn aggregation in biosensor cells than that of skin-amplified DLB strains. Further, we showed that skin-amplified PD strains induce greater number of pathological p-αSyn inclusions than skin-amplified DLB strains in biosensor cells, which is consistent with the FRET-based bioactivity. Conversely, lesser p-αSyn inclusions were induced by brain-amplified PD strains than skin-amplified PD strains. To further enhance our understanding, we thoroughly classified intracellular pathological p-αSyn inclusion morphology induced by skin- and brain-amplified αSyn strains in U251 biosensor cells. Remarkably, we found that PD and MSA skin-amplified αSyn strains impart similar p-αSyn inclusion morphologies but were distinct from skin-amplified DLB strains in U251 biosensor cells. It is intriguing that skin-derived αSyn strains are potent inducer of intracellular αSyn aggregation like the brain-derived αSyn strains when assessed in U251 biosensor cells. It has been reported that p-αSyn inclusions are detected in PD, DLB, and MSA patient skin but not in healthy skin tissue (46, 73, 74). The presence of p-αSyn in the patient skin may be attributed to the potent bioactivity of skin-amplified strains in biosensor cells. These observations suggest that skin tissue environment in synucleinopathies is conducive to generate proteopathic αSyn aggregates to be spread along the large area of the skin. Deciphering cellular mechanisms underlying this pathway could potentially have important implications for our understanding of the cell-specific environment leading to the emergence of unique αSyn strains.

To the best of our knowledge, we are the first to report the inclusion pathology induced by patient skin-amplified strains in cultured cells. In contrast, PD and DLB brain-amplified αSyn strains impart similar p-αSyn inclusion morphologies but were distinct from brain-amplified MSA strains in U251 biosensor cells. This was found to be consistent with prior studies that use patient brain homogenates in HEK293T cells or RT-QuIC-amplified αSyn strains from patient CSF (38, 55, 75). In summary, our findings were consistent with prior studies that have used patient αSyn strains either directly from brain homogenates (37, 38, 41, 55) or PMCA-amplified αSyn strains from patient brain homogenates (42, 45) or both (40, 44) to study pathological features of patient αSyn strains in cultured cells or in mice. It is noted that a prior work reported that PD brain homogenate-derived αSyn strains cannot seed A53T αSyn-YFP expressed in HEK293T cells (37). However, we showed here that skin-amplified PD αSyn strains triggered αSyn seeding and formation of detergent-insoluble and pathological p-αSyn inclusions in glioblastoma U251 biosensor cells expressing A53T αS-mTQ2 and A53T αS-mNG concomitantly. Our findings are supported by previous work that detected αSyn seeding from PD brain homogenates in HEK293T biosensor cells expressing A53T αSyn-CFP and A53T αSyn-YFP (38). Remarkably, our results showed that distinct p-αSyn morphology was induced by skin-amplified PD and DLB strains and brain-amplified PD strains, supporting the existence of potentially distinct αSyn conformers in PD and DLB patients.

An additional advantage of U251 biosensor cells is that they can be directly reprogrammed into neurons using NEUROD1 transcription factor as shown previously (57), allowing for the investigation of intracellular aggregation of αSyn in hiBNs from the same cell lineage. Unlike prior studies (57), we showed that hiBNs are physiologically functional when matured by day 48 and can generate action potentials upon current injections followed by electrophysiological recordings. Upon further characterization of our hiBNs, we found that hiBNs are glutamatergic neurons that lack PROX1 indicating that hiBNs are not granule cells. hiBNs morphologically appeared to resemble CA3 pyramidal neurons where the apical dendrite branches closer to the soma as shown in a prior study (76). Skin-amplified αSyn strains induced round or oval p-αSyn inclusions that were localized in the soma of hiBNs which is consistent with the prior work describing the localization of αSyn inclusions in PD, DLB, and MSA brains (1, 3) and studies involving αSyn inclusions in the hippocampus (2, 4, 77, 78). Skin-amplified strains were found to be neurotoxic that resulted in bulbous dystrophic neurites as previously reported while using patient PD and MSA-amplified fibrils (42). These results suggest that skin-amplified strains trigger neurodegeneration.

An open question is whether the nascent intracellular αSyn aggregates generated in U251 biosensors in response to seeding by patient-derived αSyn strains retain strain-specific properties. We thus performed RT-QuIC assay using detergent-insoluble fraction purified from U251 biosensor cells initially treated with skin or brain-amplified strains. Strikingly, we found that *in vitro* RT-QuIC seeding activity of skin-amplified PD strains is distinct from skin-amplified DLB strains and brain-amplified PD strains. This pattern is remarkably preserved in terms of FRET-based bioactivity and intracellular p-αSyn burden. This result fortified our finding that possibly distinct αSyn conformers are present in PD and DLB patients. Taken together, RT-QuIC assay of U251 biosensor cell-derived skin αS strains allows us to potentially discriminate PD and DLB strains using the patient skin.

In summary, we detected bioactive and pathologically distinct αSyn strains derived from PD, DLB, and MSA patient skin in U251 biosensor cells. We showed that the pathological p-αSyn burden triggered by skin αSyn strains in U251 biosensor cells dictate their seeding activity, thus establishing strain-specific αSyn pathology to seeding relationship.

### Advantages and limitations

U251 αSyn biosensor cells are a powerful model to interrogate disease-specific αSyn strains because these rapidly dividing cells can be used to easily detect detergent-insoluble αSyn aggregates just within 3 days of exogenous αSyn seeding and can be directly reprogrammed to postmitotic electrophysiologically functional neurons by a single transcription factor. U251 biosensor cell model can be used for studying pathophysiological mechanisms that control tissue- and strain-specific pathogenesis of PD and DLB in a high-throughput manner. Furthermore, U251 biosensor cells may be useful for early and differential diagnosis of synucleinopathies from patient skin by analyzing FRET bioactivity, inclusion morphology, and seeding activity, thereby enabling early and precise therapeutic intervention. Despite the advantages of the biosensor cell model, it is still challenging to diagnose patients with copathologies of αSyn and tau aggregates.

## Conclusions

We find that PD skin-derived αSyn strain is bioactive and pathologically distinct from DLB skin-derived αSyn strain, suggesting the existence of distinct αSyn conformers in the skin of PD and DLB patients. Like PD strains, DLB and MSA patient skin-derived αSyn strains are also bioactive and competent to induce intracellular p-αSyn inclusions in A53T αSyn biosensor cells. PD, DLB, and MSA skin-derived αSyn strains can trigger intraneuronal p-αSyn inclusions and neurodegeneration. U251 biosensor cells revealed a strain-specific relationship between intracellular pathological αSyn burden and *in vitro* seeding activity induced by patient skin-derived αSyn strains. Our findings emphasize the importance of studying tissue- and strain-specific pathogenesis of synucleinopathies.

## Experimental procedures

### Human tissue samples

Frozen tissue samples were obtained from the NIH NeuroBiobank from neuropathologically confirmed cases of PD (n = 2 from scalp skin; n = 1 from brain cortex), DLB (n = 2 from scalp skin; n = 1 from brain cortex), MSA (n = 2 from scalp skin; n = 1 from brain cortex), and non-neurodegenerative (healthy) controls (n = 1 from scalp skin; n = 1 from brain cortex). Informed consent and Institutional Review Boards approval for tissue collection and distribution were obtained by NeuroBioBank. As these tissue samples were de-identified and from deceased individuals, no further ethical approval was required.

### Tissue homogenization

Homogenates of skin (5% w/v) and brain (10% w/v) were prepared at 4 ^o^C as described previously (48). Briefly, skin tissue was first thawed and then rinsed three times in 700 μl of ice-cold 1X Dulbecco’s phosphate-buffered saline (D-PBS) (Gibco) in 1.5 ml tubes until blood was no longer visible, followed by mincing with a surgical blade into small pieces. Brain tissue was used directly. Homogenization buffer was then applied to the tissues. The buffer contained 150 mM NaCl (Invitrogen), 1% Triton X-100 (Bio-Rad), 5 mM EDTA pH 8.0 (0.5 M stock, Invitrogen), and 0.5X complete mini EDTA-free protease inhibitor cocktail (Roche Diagnostics) in 1X D-PBS. Homogenization was carried out in the presence of half the volume of tube with 0.7 mm zirconium beads (BioSpec Products) in a mini-Beadbeater-16 device (BioSpec Products) at 4 °C for five cycles with 1 min on and 3 min off. Homogenates were then centrifuged at 5000×*g* for 5 min at 4 °C. Supernatant was collected as clarified homogenate, and a 10-fold (skin homogenate) and 1000-fold (brain homogenate) dilutions were prepared in a solution containing 1X N2 (100X stock, Gibco) in D-PBS (N2/PBS). Clarified homogenate and diluted samples were immediately stored at −80 °C until further use for RT-QuIC assay.

### Amplification of αSyn strains from patient tissue homogenates by RT-QuIC

αSyn strains from skin or brain homogenates were amplified by RT-QuIC assay. Reagents for RT-QuIC assay include 0.5 M sodium phosphate pH 8.0 (J60825.AK, Thermo) stored at room temperature, 5 M NaCl (AM9759, Invitrogen) stored at 4 °C, ThT (596200, Sigma) stored at −20 °C, and HPLC-grade water (W5-4, Fisher) stored at 4 °C. RT-QuIC assay was adapted and performed as previously described (48). Eight hundred micrometer low-binding silica beads (BLBG-800-200-03, OPS Diagnostics) were rinsed in Milli-Q water and dried in a chemical hood. Each well of black 96-well plate with flat optical bottom (265301, Thermo) was loaded with six silica beads in a clean benchtop workstation. One milligram of lyophilized recombinant human αSyn (S-1001-2, lot. 051722AS, rPeptide) was reconstituted at 1 mg/ml and filtered through 0.5 ml Amicon 100 kDa filter (UFC510096, Millipore) at 10,000×*g* for 10 min at 4 °C to get rid of aggregated αSyn, if any. RT-QuIC reaction mix containing 40 mM sodium phosphate, pH 8.0, 170 mM NaCl, and 20 μM ThT in HPLC-grade water was prepared from the stock solutions as specified above and filtered through Millex-GV 0.22 μm PVDF membrane syringe filter (SLGV004SL, Millipore) into a sterile 25 ml boat while using benchtop air ionizer (963E, Static Control Solutions) to remove static charges from the work bench. Filtered recombinant αSyn was applied to the reaction mix so that the final concentration of αSyn in the reaction mix is 0.1 mg/ml. Ninety-eight microliter of the reaction mix and 2 μl of the diluted skin or brain homogenate was applied to each well of 96-well plate containing silica beads. The plate was sealed with Nunc clear sealing film (Thermo) and loaded into BMG FLUOstar Omega plate reader (BMG Labtech). The RT-QuIC reaction was run at 42 °C with 1 min double-orbital shaking at 400 rpm and 1 min rest, and optical gain was set at 1800. ThT fluorescence (448/10 nm excitation and 482/10 nm emission) was recorded every 45 min. RT-QuIC run was stopped when ThT fluorescence of healthy control samples reached a cut-off value of 50,000 RFUs. Each sample was run in triplicates and was considered positive if at least two out of three exceeded the cut-off RFU. Average ThT fluorescence was calculated for each sample regardless of whether they exceed the cut-off RFU. Raw data of ThT fluorescence were reported for each sample. To generate skin-amplified and brain-amplified αSyn fibrils, ThT was not used in the RT-QuIC reaction mix; however, amplification was simultaneously monitored in three separate wells with ThT in the reaction mix. After the RT-QuIC reaction was stopped, wells that did not contain ThT in RT-QuIC reaction mix were pooled in a 1.5 ml tube (357448, Beckman Coulter). Tubes were placed in a fixed-angle rotor (TLA-55, Beckman Coulter) and spun at 186,000*g* for 30 min in an ultracentrifuge (393315, Beckman Coulter). Pellet was carefully rinsed with 1 ml of cold D-PBS and spun again at 186,000*g* for 30 min. The pellet was suspended in 20 μl of cold D-PBS and vortexed. Purified skin- and brain-amplified fibrils were stored in 1.5 ml Protein LoBind tubes (022431081, Eppendorf) at 4 °C until further use. An aliquot of 3.5 μl of these skin- or brain-amplified αSyn fibrils were diluted in a final volume of 15 μl of 1X N2/PBS in 1.5 ml polystyrene tubes (53072, Active Motif), which was placed in a water bath preset to 4 °C in a cup-horn sonicator (Q700, 431C2, QSonica). 10 min of sonication was carried out at an amplitude of 70 with 30 s of sonication and 30 s of rest in each cycle with 250 to 350 W power output per cycle. Sonicated αSyn fibrils were briefly spun down, and 1X N2/PBS was applied to each tube to make a final volume of 35 μl. After vortexing, 2 μl of sonicated samples were applied to 98 μl of RT-QuIC reaction mix in each well with or without ThT in a 96-well plate, followed by amplification and purification as described above. These patient-derived and amplified αSyn fibrils were used in cell culture experiments. To detect *in vitro* seeding activity of αSyn strains from the cell culture supernatant, the supernatant was diluted 1000 times in 1X N2/PBS. Two microliter of diluted supernatant was then applied to 98 μl of RT-QuIC reaction mix followed by RT-QuIC as described above. To detect *in vitro* seeding activity of αSyn strains from the sarkosyl-insoluble fraction of cells, 2 μl of the purified fraction was directly applied to 98 μl of RT-QuIC reaction mix followed by RT-QuIC as described above.

### Generation of αSyn rPFFs

For *de novo* generation of αSyn rPFF, 1 mg of lyophilized human recombinant αSyn was reconstituted in 500 μl of HPLC water in 1.5 ml Protein LoBind tube and agitated at 1000 rpm for 7 days at 37 °C in a thermomixer (EP5384000012, Eppendorf). The *de novo* generated rPFFs were purified using an ultracentrifuge as described above and resuspended in 400 μl of D-PBS and stored at 4 °C until further use.

### Protein concentration

Concentration of purified skin and brain-amplified fibrils, rPFF, or cell lysates were measured by bicinchoninic acid assay using bovine serum albumin as a standard. Additionally, absorbance at 205 nm with the SCOPES method (*A*_205_) was also used to measure αSyn fibril concentration on Nanodrop (ND-ONEC-W, Thermo Fisher) from 1.5 μl of sample and using 1.5 μl of D-PBS as a blank. Samples were vortexed for 5 to 10 s just before measuring concentrations in triplicates on Nanodrop. The mean concentration from triplicates was considered as final concentration of fibrils in that sample. Protein concentrations measured using bicinchoninic acid assay and Nanodrop A_205_ were found to be comparable; henceforth, Nanodrop A_205_ was used to measure fibril concentration in each sample.

### Transmission electron microscopy

For negative staining to image skin or brain-amplified αSyn fibrils or rPFF, first samples were diluted in HPLC water to a final concentration of 0.2 μg/μl. Formvar/carbon coated 200 mesh copper grid (FCF200-Cu-50, Electron Microscopy Sciences-EMS) was placed on PELCO TEM grid-holder (Ted Pella) and glow discharged for 25 s at 20 mA using PELCO easiGlow discharge apparatus (Ted Pella). After fixing the grid in a tweezer, 5 μl of diluted sample was applied on the grid and incubated for 1 min. Excess sample was wicked off from the bottom of the grid using torn edge of Whatman filter paper. 7.5 μl of 1% uranyl acetate was applied on the grid and incubated for 45 s. Excess solution was wicked off from the edge of the grid using torn edge of Whatman filter paper. Then, grid was incubated in HPLC water drop for 30 s, and excess water was wicked off. Grid was examined using JEOL 1400 FLASH TEM at 120 kV (JEOL USA Inc), and digital images were captured using an AMT NanoSprint43L Mark-II camera (AMT Imaging).

For staining, sectioning, and imaging intracellular ultrastructure, first, U251 glioblastoma cells expressing A53T αSyn-EGFP were spun down at 400*g* for 3 min. The cell pellet was suspended in fixing solution containing 2% glutaraldehyde in 0.15 M sodium cacodylate buffer (pH 7.4) for at least 2 h at 4 °C followed by rinsing twice for 15 min each in 0.15 M sodium cacodylate buffer. Cell pellet was then treated with 1% osmium tetroxide (EMS) in 0.15 M sodium cacodylate buffer in dark at room temperature for 1 h followed by rinsing thrice for 15 min each. Pellet was then incubated in 1% low molecular weight tannic acid (Ted Pella) followed by rinsing thrice for 15 min each. Cell pellet was dehydrated through graded 50%, 80%, 95%, and 100% ethyl alcohol treatments and transitioned through 100% propylene oxide to 50% propylene oxide in embedding resin to 100% embedding resin. Cell blocks were polymerized overnight at 60 °C. Using silver to pale gold color interference, 70 to 90 nm thin sections were cut from cell blocks using a diamond knife (Diatome, EMS) on an ultramicrotome (Leica EM UC7). After drying, sections were mounted on 200 mesh copper grids and stained with 1% uranyl acetate and Reynold’s lead citrate for contrast. Grids were examined using JEOL 1400 FLASH TEM at 120 kV, and digital images were captured using an AMT NanoSprint43L Mark-II camera.

### Plasmids

Lentiviral vectors used in this study were constructed using the plasmids obtained from Addgene. Constitutive expression of transgenes in glioblastoma cells using lentiviral vectors was driven by CMV promoter. The original plasmids used in this study were as follows: pMDLg/pRRE (Addgene plasmid # 12251), pMD2.G (Addgene plasmid # 12259), and pRSV-Rev (Addgene plasmid # 12253) were a gift from Didier Trono (79). pLV-eGFP (Addgene plasmid # 36083) was a gift from Pantelis Tsoulfas (80). EGFP-alphasynuclein-A53T (Addgene plasmid # 40823) was a gift from David Rubinsztein (81). pLenti-dsRed_IRES_SNCA:EGFP (Addgene plasmid # 92195) was a gift from Huda Zoghbi (82). mTurquoise2-N1 (Addgene plasmid # 54843) was a gift from Michael Davidson and Dorus Gadella (83). ER-mNeonGreen (Addgene plasmid # 137804) was a gift from Dorus Gadella (84). EF1a_NEUROD1_P2A_Hygro_Barcode (Addgene plasmid # 120466) was a gift from Prashant Mali (85). Lentiviral vectors that were constructed are as following: CMV-A53T αSyn-EGFP-WPRE-bGH poly(A), CMV-WT αSyn-EGFP-WPRE-bGH poly(A), CMV-A53T αSyn-mTurquoise2-IRES-A53T αSyn-mNeonGreen-PGK-Z418-WPRE-bGH poly(A), CMV-A53T αSyn-mTurquoise2-IRES-PGK-Z418-WPRE-bGH poly(A), CMV-IRES- A53T αSyn-mNeonGreen-PGK-Z418-WPRE-bGH poly(A), CMV-dsRed-IRES-NEUROD1-PGK-Z418-WPRE-bGH poly(A), and CMV-dsRed-IRES-PGK-Z418-WPRE-bGH poly(A) where EGFP is enhanced green fluorescent protein, WPRE is woodchuck hepatitis virus posttranscriptional regulatory element, and bGH poly (A) is bovine growth hormone polyadenylation signal. All constructed lentiviral vectors were sequenced to confirm the integrity of transgenes.

### Cell culture

Human glioblastoma cell lines U251 (9063001, Sigma), U118, U87, U138, and DBTRG-05, as well as human embryonic kidney cell line Lenti-X HEK293T (632180, Takara) were maintained in 10 ml of growth medium consisting of Dulbecco's modified Eagle's medium (DMEM) (SH30243.01, Cytiva) with 10% fetal bovine serum (Corning) and 1% penicillin/streptomycin (P/S) (15140122, Gibco) in a 10 cm dish at 37 °C in a humidified incubator. Cells were passaged twice a week. For passaging, cells were rinsed once with D-PBS and dissociated from the dish by incubating with 1 ml of 0.05% trypsin (25-052-CV, Corning) for 3 min at 37 °C. Trypsin was deactivated with 7 ml of growth medium applied to the cells and centrifuged at 400*g* for 3 min at room temperature. Supernatant was discarded and pellet was suspended in required volume of medium for passaging in a fresh 10 cm dish with 10 ml of growth medium.

### Lentivirus production and purification

Third generation lentivirus was produced using Lenti-X HEK293T cells. Expanding Lenti-X 293T cells for lentivirus production was in part previously described (86, 87). First, cells were grown up to 60 to 70% confluency (5–8 million cells) in a 10 cm dish in growth medium. Medium was changed to 7 ml of DMEM medium, and cells were transfected with lentiviral transfer plasmid (4 μg) along with two packaging plasmids (pMDLg/pRRE: 2 μg, pRSV-REV: 0.5 μg), one envelope plasmid (pMD2.G/VSV-G: 1 μg), and 22.5 μl of 1 μg/μl of polyethylenimine in 1 ml of DMEM medium. Ratio of plasmid to polyethylenimine used was 1 to 3. Transfection was carried out for 16 h after which medium was changed back to growth medium. After 24 and 48 h in growth medium, supernatant containing lentivirus was collected and spun at 9000 rpm for 10 min to remove cell debris. 50% polyethylene glycol (w/w) was applied to the supernatant in the ratio of 1 to 8 respectively and incubated for 16 to 20 h at 4 °C to precipitate lentivirus. Supernatant was then again centrifuged at 9000 rpm for 15 min at 4 °C, and pellet was suspended in cold D-PBS, filtered through 0.45 μm PVDF filter, and centrifuged at 21,000×*g* for 1 h at 4 °C. Pellets containing lentivirus were resuspended in 20 μl of D-PBS (for 6 dishes), vortexed, aliquoted, and stored at −80 °C until further use. Lentiviral titer (transducing units/μl) was calculated by serially diluting (10 times) lentivirus on plated 50,000 U251 cells per well with 8 μg/ml of hexadimethrine bromide or polybrene (H9268-5G, Sigma) and quantifying the total number of infected cells with that lentiviral dilution per well that lie within the dynamic range of 1 to 10% infectivity.

### Generation of stable monoclonal U251 cell lines expressing fluorescently tagged αSyn

50,000 U251 cells were plated in growth medium in each well of a 24-well plate (353047, Falcon). Lentivirus expressing either WT αSyn-EGFP or A53T αSyn-EGFP or A53T αSyn-mTurquoise2-IRES-A53T αSyn-mNeonGreen was applied to each well with a multiplicity of infection equaling to 30. Lentivirus-treated cells were expanded to 10 cm dish until confluency was reached. BD FACSMelody (BD Biosciences) cell sorter was used to select 10% brightest EGFP-positive single cells (excitation 488 nm laser and emission 527/32 nm) expressing WT αSyn-EGFP or A53T αSyn-EGFP. BD FACSymphony S6 (BD Biosciences) cell sorter was used to select 5% brightest A53T αSyn-mNeonGreen-positive single cells (excitation 488 nm laser and emission 515/20 nm) coexpressing A53T αSyn-mTurquoise2 (excitation 405 nm laser and emission 450/50 nm). Unstained U251 cells and cells expressing single fluorophore were used as compensation controls to correct fluorescence spillover. Two hundred single cells were plated in 100 μl fresh growth medium per well of a 96-well plate (P96–1.5P, CellVis). Twenty colonies were selected based on growth and expanded in a 6-well plate (3516, Corning Costar). To identify a U251 monoclonal cell line out of 20 colonies that allows efficient aggregation of intracellular αSyn by exogenous αSyn rPFF, 10,000 cells from each cell clone was plated in triplicates in each well of 96-well plate, and 3 μM of sonicated rPFF was applied to the cells 1 day after plating. Four DPA of rPFF, which is 5 days *in vitro* (DIV), detergent soluble fraction was extracted and removed from the cells with cold 4% sucrose and 1% nonidet-40 (NP-40 or IGEPAL) in 1X D-PBS for 2 min. Immediately after, cells were fixed with cold 4% paraformaldehyde (PFA) (R04586–76, Sigma) and 1% NP-40 in 1X D-PBS for 15 min. The monoclonal cell line with the highest number of detergent insoluble puncta was selected, when excited with 482/25 nm LED and detected with 524/24 nm filter set in Evos M7000 fluorescence microscope (Thermo Scientific), expanded further, and frozen in liquid nitrogen with 10% DMSO in growth medium. Monoclonal cell lines were not tested for the presence of *mycoplasma*.

### Rapid in situ detection of fluorescent intracellular αSyn aggregates

Fluorescent αSyn aggregates formed intracellularly by seeding with rPFF were rapidly detected either in U251 cells expressing WT αSyn-EGFP or A53T αSyn-EGFP or U251 biosensor cells expressing A53T αSyn-mTurquoise2-IRES-A53T αSyn-mNeonGreen. Culture supernatant was discarded, and cells were treated with cold 4% sucrose and 1% NP-40 in 1X D-PBS for 2 min. The sucrose-NP-40 solution was discarded, and cells were immediately fixed with cold 4% PFA and 1% NP-40 in 1X D-PBS for 15 min. While the cells were being fixed, fluorescent αSyn aggregates resembling ring-like structures or puncta were easily detected in Evos M7000 when excited with 482/25 nm LED and detected with 524/24 nm filter set. After fixing, cells were rinsed once with 100 μl of D-PBS and stored in 200 μl of D-PBS containing 0.03% sodium azide (storage buffer) at 4 °C. Intracellular fluorescent αSyn aggregates were more clearly visible in storage buffer.

### Intracellular αSyn aggregation induced by exogenous proteopathic seeds

For seeding with αSyn rPFF without lipofectamine, monoclonal U251 cell line expressing WT αSyn-EGFP or A53T αSyn-EGFP, 10,000 cells were plated in each well of 96-well plate in growth medium. Next day, 100% medium was changed to 90 μl of fresh medium. Required volume of rPFF, at least 3 μl in final volume of 15 μl D-PBS, was prepared in 1.5 ml polystyrene tubes that were placed in a water bath preset to 4 °C in a cup-horn sonicator (Q700, 431C2, QSonica). Ten minutes of sonication was set and carried out at an amplitude of 70 with 30 s of sonication and 30 s of rest with 250 to 350 W power output per cycle. Ten microliter of sonicated rPFF per well were applied to the cells within 2 h of sonication so that the final concentration per well was 3 μM (4320 ng), which was equivalent to 432 pg per cell. D-PBS vehicle or αSyn monomer equivalent in D-PBS was used as a negative control.

For monoclonal biosensor U251 cell line expressing A53T αSyn-mTurquoise2 and A53T αSyn-mNeonGreen, 30,000 cells were plated in each well of 96-well plate in growth medium. Next day, 100% medium was changed to 120 μl of fresh medium. Required volume of skin or brain-amplified fibrils, at least 3 μl in final volume of 15 μl D-PBS, was prepared in 1.5 ml polystyrene tubes and sonicated as described above. Sonicated patient-amplified αSyn fibrils were diluted further in cold D-PBS as desired. 6% (v/v) lipofectamine 2000 (11668027, Invitrogen) in Opti-MEM (31985070, Gibco) was incubated for 5 min at room temperature. Equal volume of sonicated patient-amplified αSyn fibrils was applied to 6% lipofectamine drop-wise and gently mixed by triturating 4 to 5 times. Lipofectamine-αSyn fibril mix was incubated for at least 30 min at room temperature. Thirty microliter of lipofectamine-αSyn fibril mix was applied to each well of cells containing 120 μl of growth medium. Final concentration of lipofectamine 2000 in each well of 96-well plate was 0.6% in 150 μl final medium volume. Final concentration of patient-amplified αSyn fibrils per well of 96-well plate was 100 nM (216 ng), which was equivalent to 7.2 pg of patient fibrils per cell. Concentrations of patient-amplified αSyn fibrils and lipofectamine were kept constant while using a 6-well plate, wherein, 180 μl of Lipofectamine-αSyn fibril mix was applied to 200,000 cells in a final medium volume of 1 ml per well resulting in 100 nM (1440 ng) of patient-derived fibrils per well, which was also equivalent to 7.2 pg of patient fibrils per cell. Equimolar concentrations of lipofectamine-αSyn monomer and lipofectamine-rPFF mix were used as controls. Experiments were carried out until desired time-point with medium replenishments, and cells were processed accordingly for further analyses by RT-QuIC assay, immunofluorescence, immunoblotting, and FRET-Flow cytometry.

### FRET-flow cytometry

100 nM of skin- or brain-amplified αSyn fibrils with lipofectamine were applied on 30,000 monoclonal biosensor U251 cells in a well of a 96-well plate as described above. After 4 DPA of αSyn fibrils, supernatant was discarded, and cells were rinsed once with 50 μl D-PBS per well. Cells were incubated at 37 °C with 50 μl of 0.05% trypsin for 3 min that was deactivated with 130 μl of growth medium. Dissociated cells were transferred into 96-well U-bottom plate (7007, Corning) and spun at 1,000*g* for 3 min. Medium was carefully discarded, and cells were suspended and fixed in 50 μl of cold 2% PFA in D-PBS for 10 min at room temperature. After fixing, plate was spun at 1,000×*g* for 3 min, and cell pellet was resuspended in 105 μl of cold FACS buffer containing 2% fetal bovine serum and 1 mM EDTA in D-PBS. Seventy microliter of biosensor cell suspension was aspirated by Attune NXT flow cytometer (ThermoFisher), and FlowJo v10.10.0 software was used to quantify percent of cells positive for FRET (FRET percentage) and efficiency of αSyn oligomerization in cells (FRET efficiency). Median fluorescence intensities of mTurquoise2 (donor) and FRET were measured by exciting U251 cells at 405 nm and detecting emission at 440/50 nm and 530/30 nm respectively. Median fluorescence intensity (MFI) of mNeonGreen (acceptor) was measured by exciting cells at 488 nm and detecting emission at 530/30 nm. MFI ratio of donor to acceptor in monoclonal biosensor cell line was found to be 1 to 8.9 ± 0.3 SEM (N = 12). To quantify FRET, gating strategy was adapted from previously described study (88). Bleedthrough of mNeonGreen into FRET channel was negligible in these cells. A bivariate plot of mTurquoise2 and FRET was created. Lipofectamine-αSyn monomer–treated cells were used to adjust gate to exclude single cell population that do not contain αSyn aggregates and were thus FRET negative. This gate on the bivariate plot allowed us to quantify FRET-positive cells with sensitized acceptor emission at 530/30 nm by donor excitation at 405 nm occurring due to αSyn oligomerization and formation of intracellular αSyn aggregates. Integrated MFI was calculated by the product of FRET percentage and FRET MFI. Each data point resulted from 20,000 to 25,000 single cells analyzed out of 100,000 to 110,000 total cells per well.

### Immunofluorescence

100 nM of skin- or brain-amplified αSyn fibrils with lipofectamine were applied on 30,000 monoclonal biosensor U251 cells in a well of a 96-well plate as described above. After 4 DPA of αSyn fibrils, supernatant was discarded, and cells were rinsed once with 50 μl D-PBS. Cells were then fixed in 100 μl of cold 4% PFA for 15 min at room temperature. Cells were rinsed twice with 100 μl of cold D-PBS and stored in 200 μl of D-PBS with 0.03% sodium azide at 4 °C. Immunofluorescence was performed as previously described (89, 90). Storage solution was discarded, and cells were blocked in blocking buffer containing 5% normal donkey serum (0030–01, Southern Biotech) in 0.3% NP-40 in 1X D-PBS (rinse buffer) for 20 min at room temperature in dark. Primary antibody was diluted in fresh blocking buffer and incubated with cells at room temperature for 1 h in dark. Cells were rinsed three times in 100 μl of rinse buffer followed by incubating with secondary antibody diluted in fresh blocking buffer at room temperature for 1 h in dark. To counterstain nuclei (if required), 50 μl of 4 μg/ml of DAPI (D9542, Sigma) in D-PBS was applied to the cells and incubated for 3 min after discarding secondary antibody solution. Cells were then rinsed three times in 100 μl of rinse buffer and once with 1X D-PBS followed by storing in 200 μl of fresh D-PBS. Plate was then sealed with Nunc clear sealing film and was stored with lid closed at 4 °C. Primary antibodies used were as following: rabbit anti-p-αSyn (1:500 dilution, Abcam Cat# ab51253, RRID:AB_869973), mouse anti-TUJ1 (1:1000, R&D Systems Cat# MAB1195, RRID:AB_357520), mouse anti-NeuN (1:500, Sigma-Aldrich Cat# MAB377, RRID:AB_2298772), and mouse anti-NEUROD (1:250, Santa Cruz Biotechnology Cat# sc-46684, RRID:AB_671759). Secondary antibody used were as following: donkey anti-rabbit IgG Alexa fluor 647 (1:500, Thermo Fisher Scientific Cat# A-31573, RRID:AB_2536183), donkey anti-mouse IgG Alexa fluor 555 (1:500, Thermo Fisher Scientific Cat# A-31570, RRID:AB_2536180), and donkey anti-mouse IgG Alexa fluor 647 (1:500, Thermo Fisher Scientific Cat# A-31571, RRID:AB_162542). Specificity of primary antibodies were tested using positive and negative controls. Specificity of secondary antibodies were tested on samples that were not treated with primary antibodies (data not shown). All primary and secondary antibodies used in this study were specific to their expected target proteins.

### Fluorescence microscopy and imaging

Fluorescent images were acquired either by Nikon A1R-HD Confocal T-2 Eclipse Inverted Microscope with Nis Elements software using Gasp PMT Detectors (Fig. 1, *C*, *H*, and *I*) or Leica Stellaris 5 Confocal DMI8 Inverted Microscope with LAS-X Software using HyD Detectors (Fig. 3) or BioTek Cytation 3 (Fig. 1*A*) and C10 spinning-disk confocal imaging reader with Gen5 software (Agilent) (Fig. 4). On Nikon A1R-HD Confocal, FRET was excited at 409 nm, and its emission was detected by 538/42 filter set. mTurquoise2 was excited at 409 nm, and its emission was detected by 450/25 filter set. DAPI was excited at 409 nm, and its emission was detected by 450/50 filter set. EGFP and mNeonGreen were excited at 489 nm, and their emission was detected by 525/50 filter set. Alexa Fluor 647 bound to p-αSyn antibody was excited at 638 nm, and its emission was detected by 700/75 nm filter set. On Leica Stellaris 5 Confocal microscope, FRET was excited at 405 nm, and its emission was detected by 532/37 filter set. mTurquoise2 was excited at 405 nm, and its emission was detected by 455/41 filter set. Alexa Fluor 647 bound to p-αSyn antibody was excited at 653 nm, and its emission was detected by 709/100 nm filter set. mNeonGreen was excited at 488 nm, and its emission was detected by 525/50 filter set. To counterstain nuclei, SYTOX orange was excited at 553 nm, and its emission was detected by 600/84 filter set. On BioTek Cytation C10 spinning-disk confocal reader, DAPI was excited at 377 nm, and its emission was detected by 447/60 nm filter set. FRET was excited using 400/40 nm LED, and its emission was detected by 550/49 nm filter set. EGFP was excited at 472 nm, and its emission was detected by 525/39 nm filter set. SYTOX orange was excited at 556 nm, and its emission was detected by 600/37 filter set. Alexa Fluor 647 bound to p-αSyn antibody was excited at 635 nm, and its emission was detected by 685/40 nm. When live cells were analyzed on Evos M7000 fluorescence microscope, FRET was excited by 445/45 nm LED, and its emission was detected by 542/27 nm filter set. mNeonGreen was excited by 482/25 nm LED, and its emission was detected by 524/24 nm filter set. dsRed was excited by 531/40 nm LED, and its emission was detected by 593/40 filter set.

### Purification of intracellular αSyn fibrils from U251 biosensor cells

Skin- or brain-amplified αSyn fibrils (300 nM) with lipofectamine were applied on 200,000 monoclonal biosensor U251 cells in a 6-well plate as described above. After 5 DPA of αSyn fibrils, supernatant was discarded, and 250 μl of cold extraction buffer containing 50 mM Tris pH 7.4 at 25 °C (T-2663, Sigma), 800 mM NaCl, 1% NP-40, 10% sucrose (w/v), 1X EDTA-free protease and phosphatase inhibitor (A32961, Thermo), 1 mM EDTA, and 1 mM DTT was applied on the cells and incubated for 2 min at room temperature to extract detergent-soluble proteins. The applied extraction buffer was saved as detergent soluble fraction. Then, NP-40 in extraction buffer was replaced by 2% sarkosyl (61747, Sigma) and was applied to the cells. Cells were scrapped in 300 μl of sarkosyl buffer, and plate was nutated at room temperature for 1 h. Cell homogenate was transferred to 1.5 ml tube, nutated at 37 °C for 15 min, and then sonicated at amplitude 30 for 30 s at 4 °C. Homogenate was spun at 10,000*g* for 10 min at 4 °C to pellet out cell debris. Supernatant was collected and spun at 186,000*g* for 30 min at 4 °C. Supernatant was collected and mixed with detergent soluble fraction that was collected earlier. Pellet was rinsed with 800 μl of cold D-PBS followed by spinning at 186,000*g* for 30 min. Pellet was resuspended in 15 μl of cold D-PBS. Protein concentration was measured using Nanodrop A_205_ as previously described. For RT-QuIC analysis and amplification of U251 cell-derived αSyn fibrils, the detergent-insoluble fraction was sonicated at amplitude 70 for 10 min with 30 s sonication and 30 s rest at 4 °C as previously described. Sonicated fraction was then used for RT-QuIC amplification or stored at −80 °C for later use. For immunoblotting, 4X LDS sample buffer (B0007, Novex) with 200 mM DTT was applied to the detergent insoluble fraction (1.2X LDS-DTT sample buffer final concentration), heated to 95 °C for 5 min, and stored at −20 °C for further analysis.

### Immunoblotting and densitometric analysis

Immunoblotting was performed as previously described (86, 89). 4X LDS sample buffer with 200 mM DTT was applied to detergent soluble or insoluble fraction or clarified cell lysate (1.2X LDS-DTT sample buffer final concentration). Samples were heated to 95 °C for 5 min. Samples equivalent to 5 μg total protein amount were loaded on 4 to 12% bis-Tris protein gel (NP0323BOX, Fisher) and run in 1X Tris-MES-SDS running buffer (pH 7.3) containing 50 mM Tris base, 50 mM MES, 0.1% SDS, and 1 mM EDTA at 100 V for 1.5 h. Protein bands were transferred to 0.45 μm pore-sized PVDF membrane (IPVH00010, Immobilon-P, Millipore) in cold 1X Tris-glycine transfer buffer containing 25 mM Tris, 192.4 mM glycine, and 20% methanol at 20 V (2.5 A limit) for 1 h by a semi-dry method using Trans-Blot Turbo transfer system (Bio-Rad). After transfer, PVDF membrane was rinsed once in 0.1% Tween-20 in D-PBS (wash buffer) and blocked in 5% BSA in 0.1% Tween-20/D-PBS (block buffer) for 20 min. Primary antibody was diluted in block buffer and applied to PVDF membrane that was incubated at 4 °C in dark for 16 h. PVDF membrane was then rinsed three times in wash buffer. Secondary antibody coupled to horseradish peroxidase was diluted in block buffer and then applied to PVDF membrane that was incubated at room temperature in dark for 1 h. PVDF membrane was again rinsed three times in wash buffer. ECL (RPN2232, Cytiva) was used for chemiluminescent detection of protein bands using ChemiDoc imaging system (Bio-Rad). Densitometric analysis of protein bands was performed using ImageJ2, version 2.16.0/1.54p. First, all protein bands at around expected molecular weight range from different treatments on the same membrane were selected. The selected molecular weight range was kept constant for all replicates. Then, band intensities were converted to peaks. Peak area of each protein band was integrated and calculated. The integrated band intensity of p-αSyn was normalized to the integrated band intensity of total αSyn in each treatment and reported as a percentage. Primary antibodies used were as follows: rabbit anti-αSyn (1:1000 dilution, Abcam Cat# ab138501, RRID:AB_2537217), rabbit anti-p-αSyn (1:1000), and mouse anti-GAPDH (1:10,000, Proteintech Cat# 60004-1-Ig, RRID:AB_2107436). Secondary antibodies used were as follows: donkey anti-rabbit HRP (1:10,000, Thermo Fisher Scientific Cat# SA1-200, RRID:AB_325994) and donkey anti-mouse HRP (1:10,000, Thermo Fisher Scientific Cat# SA1-100, RRID:AB_325993). All primary and secondary antibodies used in this study were specific to their expected target proteins.

### Quantification and classification of p-αSyn aggregate morphology

Pathological αSyn (p-αSyn positive) aggregates in biosensor U251 cells and induced biosensor neurons were quantified, and their morphology was assessed postfixation in 4% PFA. 96-well plate was loaded into BioTek Cytation C10 spinning-disk confocal imaging reader (Agilent), and confocal images were captured by Hamamatsu scientific CMOS Orca camera and automatically deconvoluted by Gen5 software (Agilent). Biosensor U251 cell nuclei were counterstained with 0.5 μM of SYTOX Orange (S11368, Invitrogen) in D-PBS after imaging p-αSyn aggregates, whereas mouse anti-TUJ1 antibody was used to counterstain induced biosensor neurons simultaneously with rabbit anti-p-αSyn antibody. Nuclei and cell bodies (soma with nucleus) were quantified automatically using Gen5 software with optimized settings. Intracellular p-αSyn aggregates in biosensor U251 cells were classified into four classes of phenotypic morphologies based on MFI and size of the p-αSyn aggregates. They were named diffuse speckles, diffuse inclusions, compact speckles, and compact inclusions. MFI of 8000 to 14,999 was termed as diffuse, whereas MFI ≥15,000 was termed as compact. When a p-αSyn aggregate cover a smaller area of 0.5 to 4.5 μm in a cell, it was termed as a speckle, whereas, when it covers a larger area of 4.6 to 35 μm, it was termed as an inclusion. p-αSyn aggregates in biosensor cells that meet the above criteria were quantified automatically using Gen5 software. In U251 biosensor cell-derived induced neurons, only those p-αSyn aggregates that overlap specifically with TUJ1-positive neuronal cell bodies and meet the above criteria were quantified automatically. Length of p-αSyn aggregates in neuronal cell bodies were calculated automatically using Gen5 software by drawing a straight line across the farthest points in those aggregates. Images were acquired from four different locations in a well of 96-well plate. Precisely, same location in all wells were used for image acquisition. Total area of all locations in a well amounted to 3.86 mm^2^ for analyzing biosensor cells and 8.06 mm^2^ for induced biosensor neurons. Each data point was represented as mean ± SEM of all four images from each well. Quantified p-αSyn aggregates were normalized to the area and number of nuclei or cell bodies from each image in each well. Proportion of each morphology for each disease group in biosensor cells was quantified after normalization and plotted against each morphological phenotype of speckles and inclusions. From biosensor cells, each data point resulted from 5000 to 10,000 cells with 1500 to 14,000 p-αSyn speckles and inclusions analyzed. From induced biosensor neurons, each data point resulted from 180 to 700 neuronal cell bodies analyzed.

### Generation of U251-induced biosensor neurons

Generation of induced neurons from U251 biosensor cells was adapted from a prior study (57). Monoclonal biosensor U251 cells were transduced with lentivirus to deliver NEUROD1 and dsRed or dsRed alone (control) with a multiplicity of infection equaling to 15. Lentivirus that delivered dsRed only was used a control for reprogramming. Lentivirus was applied to cell suspension containing growth medium with 8 μg/ml polybrene for 15 min in 37 °C water bath with intermittent mixing. Two thousand seven hundred cells in 200 μl of growth medium were plated in each well of a 96-well plate, coated with Matrigel (354277, Corning), which was diluted 500 times in DMEM. After 3 DPI, which was considered DIV 0, 100% of growth medium was replaced with neuronal differentiation medium containing DMEM/F12/Neurobasal (2:2:1) (SH30243 Cytiva, 51445C Sigma, 12348017 Gibco), 0.8% N2 (17502048, Gibco), 0.4% B-27 plus (A3653401, Gibco), with small molecules 1 μM dorsomorphin (P5499, Sigma), 10 μM forskolin (S2449, Selleck Chemicals), with growth factors 20 ng/ml brain-derived neurotrophic factor (450-02, Gibco), 20 ng/ml glial cell-line derived neurotrophic factor (450-10, Gibco), 20 ng/ml NT3 (450–03, Gibco), and 1% penicillin/streptomycin. 50% neuronal medium with freshly added small molecules and growth factors was changed every other day until 21 DPI after which medium replacement was done twice a week. Lentivirus transduction was reported by dsRed-positive cells. NEUROD1-expressing cells develop long neuritic extensions emerging from the cell body causing dsRed levels to diffuse resulting in lower dsRed signal intensity. Control group of cells do not express NEUROD1 and thus do not develop long neurites from the cell body thereby accumulating dsRed signal with relatively higher intensity in the cell body. 100 nM of skin or brain-amplified αSyn fibrils with lipofectamine was applied on DPI 34 (DIV 31), as described above, and replaced with 200 μl of fresh neuronal medium after 16 h αSyn monomer equivalent with lipofectamine was used as a control. After 14 DPA of patient fibrils (DPI 48, DIV 45), hiBNs were processed for desired analyses.

### Electrophysiology of induced biosensor neurons

Fourteen thousand monoclonal U251 biosensor cells transduced with lentivirus delivering NEUROD1 and dsRed were plated on Matrigel-coated coverslips (6330029, Carolina Science and Math). At DPI 48 (DIV 45), these coverslips were transferred to a chamber on a BX51WI Olympus microscope and superfused with recording solution containing 125 mM NaCl, 2.5 mM KCl, 1.25 mM Na_2_HPO_4_, 1 mM MgCl_2_, 2 mM CaCl_2_, 25 mM D-glucose, and 25 mM NaHCO_3_ bubbled with 5% CO_2_/95% O_2_. dsRed reporter was used to track induced biosensor neurons and were also visualized using epifluorescence illumination with Texas Red or FITC filter sets. Fire-polished borosilicate glass electrodes (BF150–86–10, Sutter Instrument) with resistance of 4 to 6 MΩ when filled with intracellular solution were mounted on the headstage (CV-7B) of a Multiclamp 700A amplifier (Molecular Devices). For whole-cell recordings, patch pipettes were filled with 135 mM K-gluconate, 2 mM MgCl_2_, 10 mM HEPES, 3 mM KCl, 0.5 mM Na-GTP, 2 mM Mg-ATP, 10 mM phosphocreatine, and 0.1 mM EGTA, pH 7.3, and 310 mOsm. Recordings were made at room temperature, and bridge balance was automatically adjusted in current-clamp recordings to compensate series resistance (<25 mΩ). Currents were sampled at 10 kHz and filtered at 2 kHz (Digidata 1440A; Molecular Devices) using PClamp 10 software (Molecular Devices). Action potentials were elicited in current clamp by increasing current steps measured during 500 ms. Sodium currents induced by increasing voltage steps (300 ms) were measured in cells held at −80 mV. Data were analyzed with Clampfit (Molecular Devices).

### Statistical analyses

All statistical analyses were performed using GraphPad Prism v10.4.1 (GraphPad). All data were examined for normality by Shapiro–Wilk test. For non-normally distributed data, unpaired two-tailed Mann–Whitney’s test was used for two groups, and Kruskal–Wallis with Dunn’s multiple comparison test was used for three or more groups. All data were also examined for differences in variance. For significantly different variance among groups, unpaired two-tailed Student’s *t* test with Welch’s correction was used for two groups, and Brown–Forsythe and Welch ANOVA with Dunnett’s T3 multiple comparison test was used for three or more groups. For normally distributed data with no significant differences in variance, unpaired two-tailed Student’s *t* test was used for two groups, and one-way or two-way ANOVA with Tukey’s multiple comparison test was used for three or more groups. Null hypothesis was rejected if *p* value was < 0.05, which was therefore considered statistically significant. Statistical powers of comparisons were analyzed by G∗Power 3.1 (91), and values were reported. Following settings were used: *t* tests, two-tailed, difference between two independent means (two groups), *post hoc* power analysis, and an α error probability of 0.05. The effect size d was determined by means and standard deviation of the two groups that were being analyzed, and the sample size of the two groups were provided. Using the settings with determined and provided values, the power (1-ß error probability) of the statistical assessment was calculated. Statistical power of the respective statistical analysis was accepted to be reasonable if it was greater than 0.8.

## Data availability

Data are provided within the manuscript or supporting information files.

## Supporting information

This article contains supporting information.

## Conflict of interest

The authors declare that they have no conflicts of interest with the contents of this article.
