# Supplementary material for: Skin-derived α-synuclein strains from PD, DLB, and MSA induce distinct intracellular pathology and neurodegeneration[image]
Source: J Biol Chem. 2025 Dec 8;302(1):111005. doi: 10.1016/j.jbc.2025.111005 (PMC12805178; doi:10.1016/j.jbc.2025.111005)
Supplement: Supporting information [file mmc1.pdf]

Supporting Information

Skin-derived α-synuclein strains from PD, DLB, and MSA induce distinct intracellular pathology and neurodegeneration

Anupam Raina[1*], Wen Wang[1], Jose Carlos Gonzalez[3], Xiaohui Yan[3], Linda Overstreet-Wadiche[3], Jacques I. Wadiche[3], Chun-Li Zhang[2*], and Shu G. Chen[1*]

1. Department of Pathology, University of Alabama at Birmingham, Birmingham, Alabama, USA.
2. Department of Molecular Biology, University of Texas Southwestern Medical Center, Dallas, Texas, USA.
3. Department of Neurobiology, University of Alabama at Birmingham, Birmingham, Alabama, USA.

*Corresponding authors:

Anupam Raina, araina@uab.edu, ORCID: 0000-0001-9995-9219.
Chun-Li Zhang, chun-li.zhang@utsouthwestern.edu, ORCID: 0000-0002-2639-4605.
Shu G. Chen, shuchen@uab.edu, ORCID: 0000-0001-7180-3001.

List of materials:

Fig. S1
Fig. S2
Fig. S3
Fig. S4
Fig. S5
Fig. S6
Fig. S7

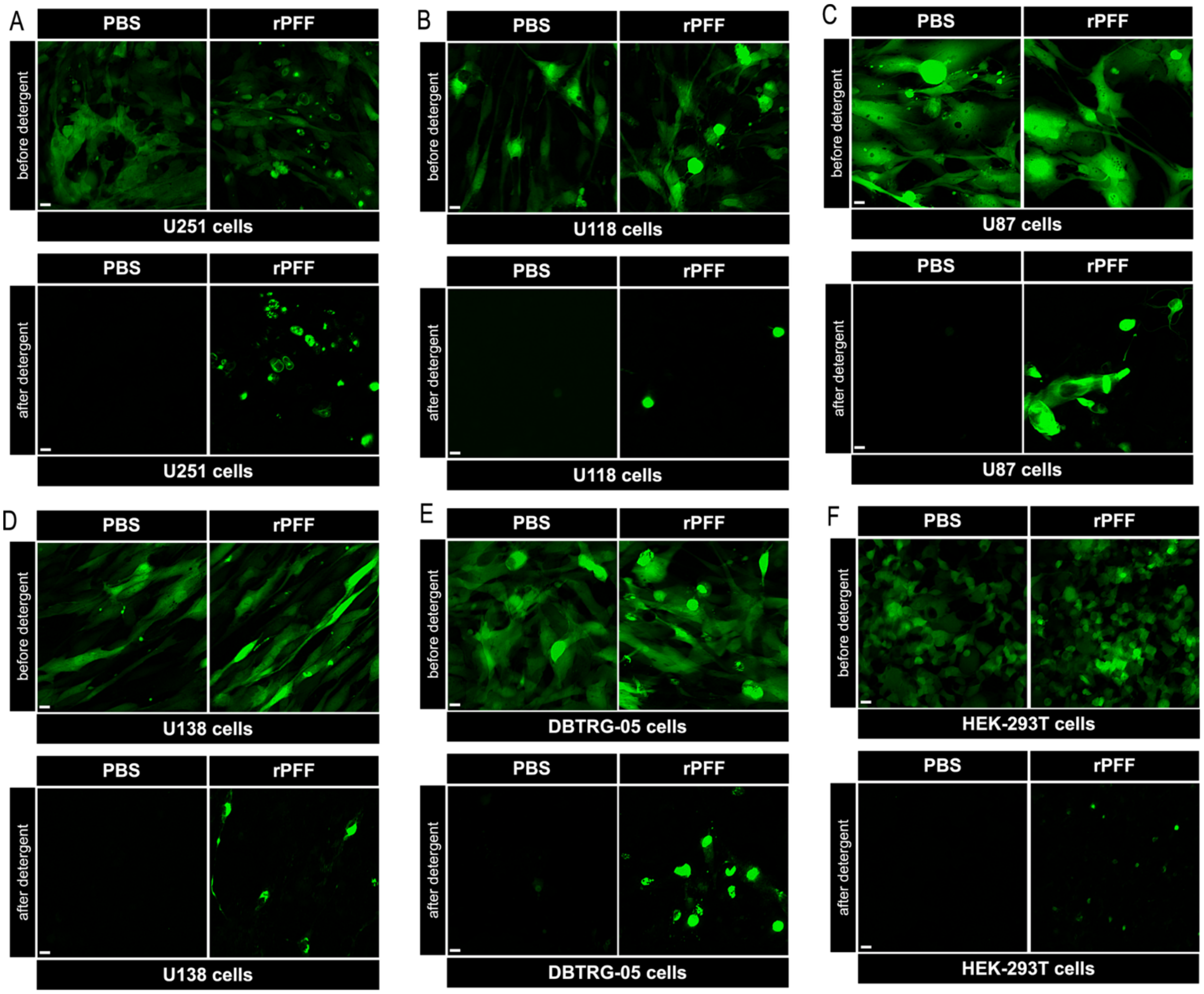

Fig. S1 Application of detergent on live glioblastoma cells reveals detergent-insoluble intracellular αSyn-EGFP aggregates.

(**A-F**) Representative live images before and after detergent treatment reveals total αSyn-EGFP and detergent-insoluble αSyn-EGFP inclusions in the indicated cell lines after application of WT αSyn rPFF or D-PBS at DPA 3 (DIV/DPI 7). Scale bars: 10µm. DIV: days *in vitro*, DPA: days post-application of rPFF, DPI: days post-infection with lentivirus.

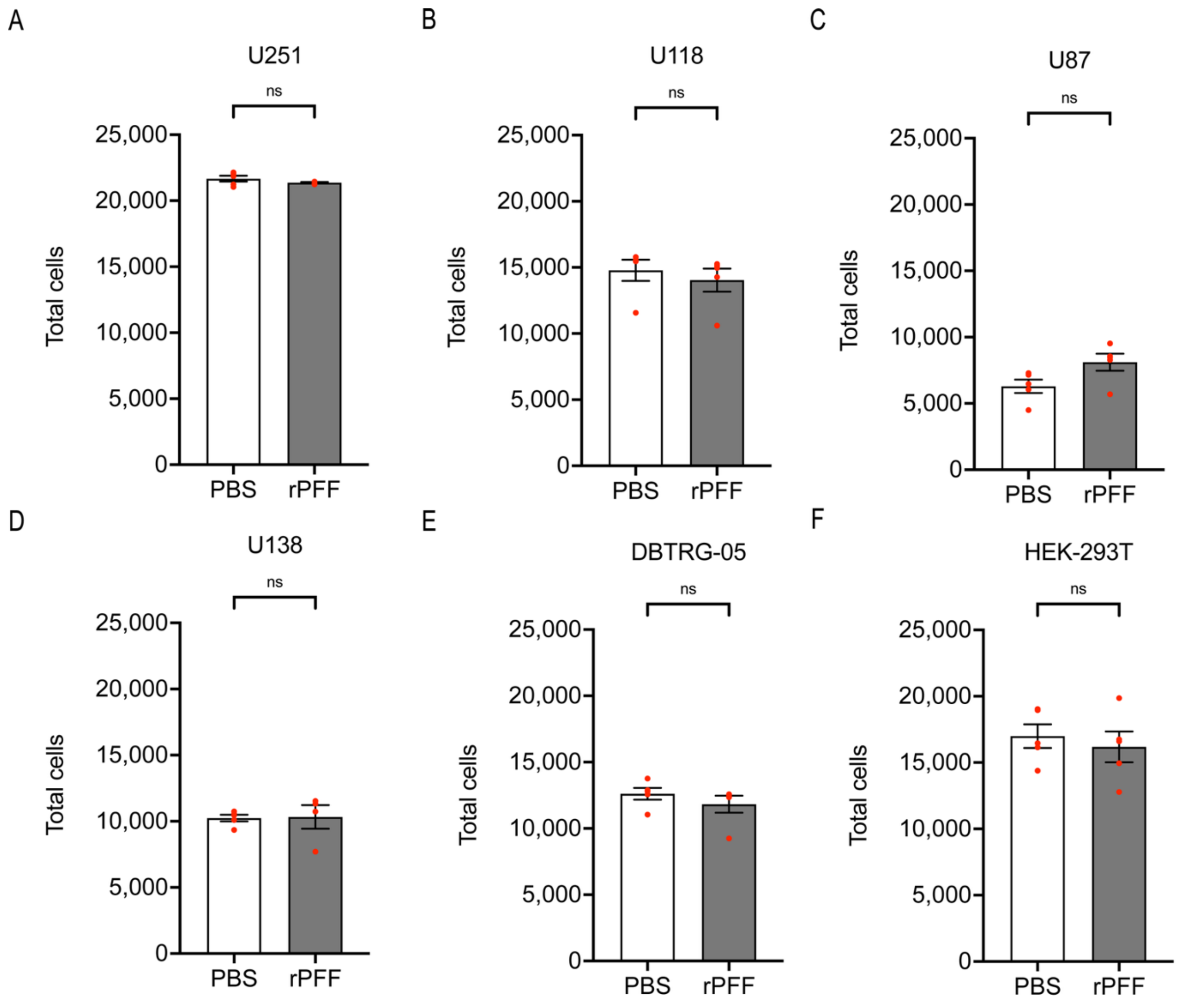

Fig. S2 Total glioblastoma cells counted by high-content imaging.
(**A-F**) Quantification of total cells positive for DAPI following PBS and rPFF treatments in the indicated cell lines at DPA 3 (DIV/DPI 7). Data are presented as mean ± SEM and each dot corresponds to a well; N=4-5 per group.
Statistical analysis was performed by unpaired two-tailed Student's t-test with (A, D) or without Welch's correction (C, F) or unpaired two-tailed Mann-Whitney test (B, E).

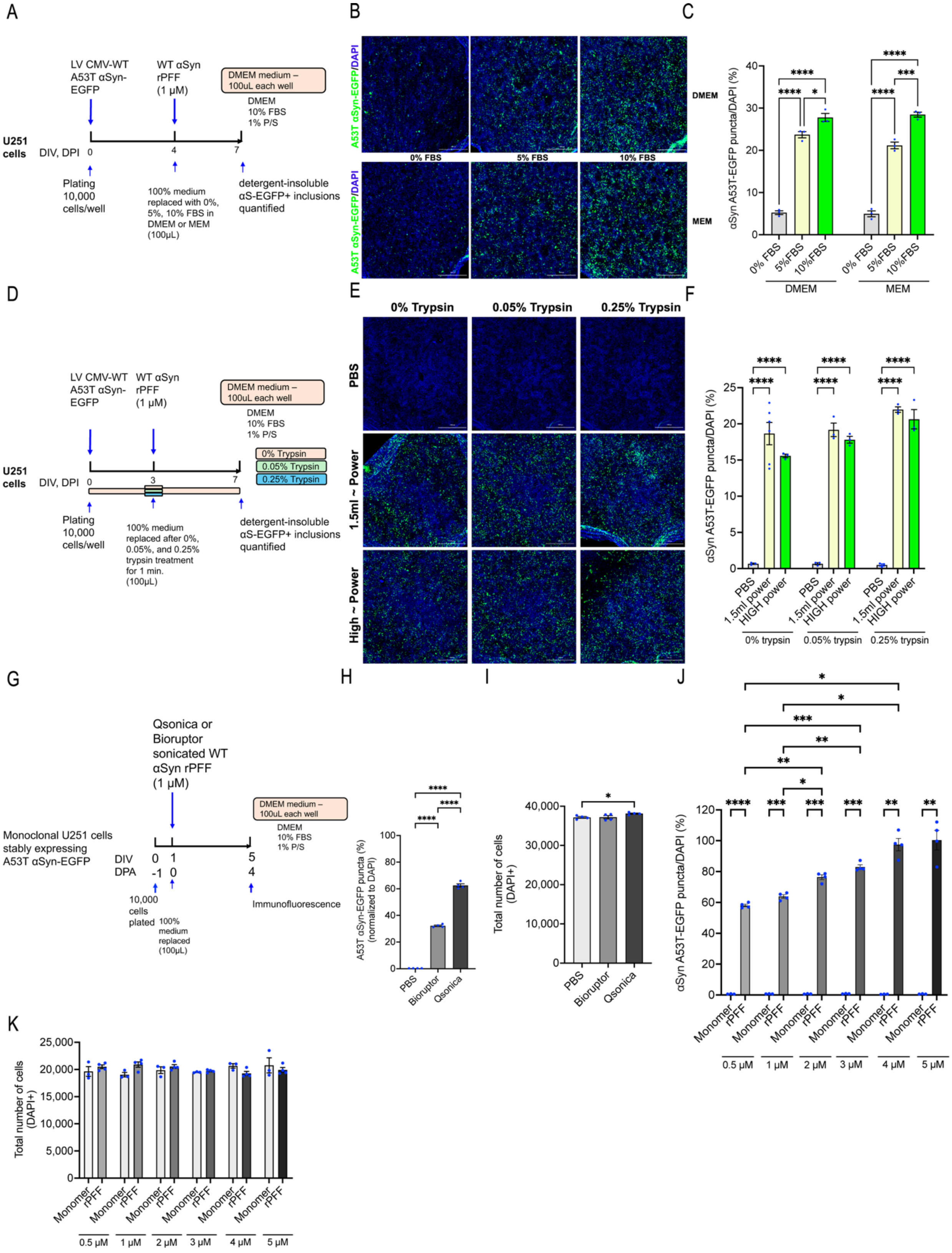
A
LV CMV-WT A53T αSyn-EGFP
WT αSyn rPFF (1 μM)
DMEM medium – 100uL each well
DMEM 10% FBS 1% P/S
U251 cells
DIV, DPI
0
4
7
Plating 10,000 cells/well
100% medium replaced with 0%, 5%, 10% FBS in DMEM or MEM (100μL)
detergent-insoluble αS-EGFP+ inclusions quantified
B
A53T αSyn-EGFP/DAPI
0% FBS
5% FBS
10% FBS
DMEM
MEM
C
αSyn A53T-EGFP puncta/DAPI (%)
0% FBS
5%FBS
10%FBS
DMEM
MEM
D
LV CMV-WT A53T αSyn-EGFP
WT αSyn rPFF (1 μM)
DMEM medium – 100uL each well
DMEM 10% FBS 1% P/S
0% Trypsin
0.05% Trypsin
0.25% Trypsin
U251 cells
DIV, DPI
0
3
7
Plating 10,000 cells/well
100% medium replaced after 0%, 0.05%, and 0.25% trypsin treatment for 1 min. (100μL)
detergent-insoluble αS-EGFP+ inclusions quantified
E
0% Trypsin
0.05% Trypsin
0.25% Trypsin
PBS
1.5ml ~ Power
High ~ Power
F
αSyn A53T-EGFP puncta/DAPI (%)
PBS
1.5ml power
HIGH power
0% trypsin
0.05% trypsin
0.25% trypsin
G
Qsonica or Bioruptor sonicated WT αSyn rPFF (1 μM)
DMEM medium – 100uL each well
DMEM 10% FBS 1% P/S
Monoclonal U251 cells stably expressing A53T αSyn-EGFP
DIV
DPA
0 1 5
-1 0 4
10,000 cells plated
100% medium replaced (100μL)
Immunofluorescence
H
A53T αSyn-EGFP puncta (%) (normalized to DAPI)
PBS
Bioruptor
Qsonica
I
Total number of cells (DAPI+)
PBS
Bioruptor
Qsonica
J
αSyn A53T-EGFP puncta/DAPI (%)
Monomer
rPFF
0.5 μM
1 μM
2 μM
3 μM
4 μM
5 μM
K
Total number of cells (DAPI+)
Monomer
rPFF
0.5 μM
1 μM
2 μM
3 μM
4 μM
5 μM

Fig. S3 Optimizing U251 cell culture conditions for enhancing naked seeding with αSyn rPFF.

(**A, D, G**) Experimental design showing the timelines of lentivirus and αSyn rPFF application with medium changes and trypsin pre-treatment.

(**B, E**) Representative images showing detergent-insoluble A53T αSyn-EGFP inclusions (green) and cell nuclei (DAPI) in the indicated cell culture conditions.

(**C, F, H, I, J, K**) Quantification of detergent-insoluble A53T αSyn-EGFP inclusions and total cells for the indicated cell culture and αSyn rPFF sonication conditions. Data are presented as mean ± SEM, and each dot corresponds to data analyzed from 20,000-36,000 cells on average in a well; N=3-5 per group.

Statistical analysis was performed by ordinary one-way ANOVA with Tukey's multiple comparison post-hoc test or by Brown-Forsythe and Welch ANOVA with Dunnet's T3 multiple comparison test (I, J) and shown as *p<0.05, **p<0.01, ***p<0.001, ****p<0.0001. Statistical power is > 0.90 between groups with p-value < 0.05.

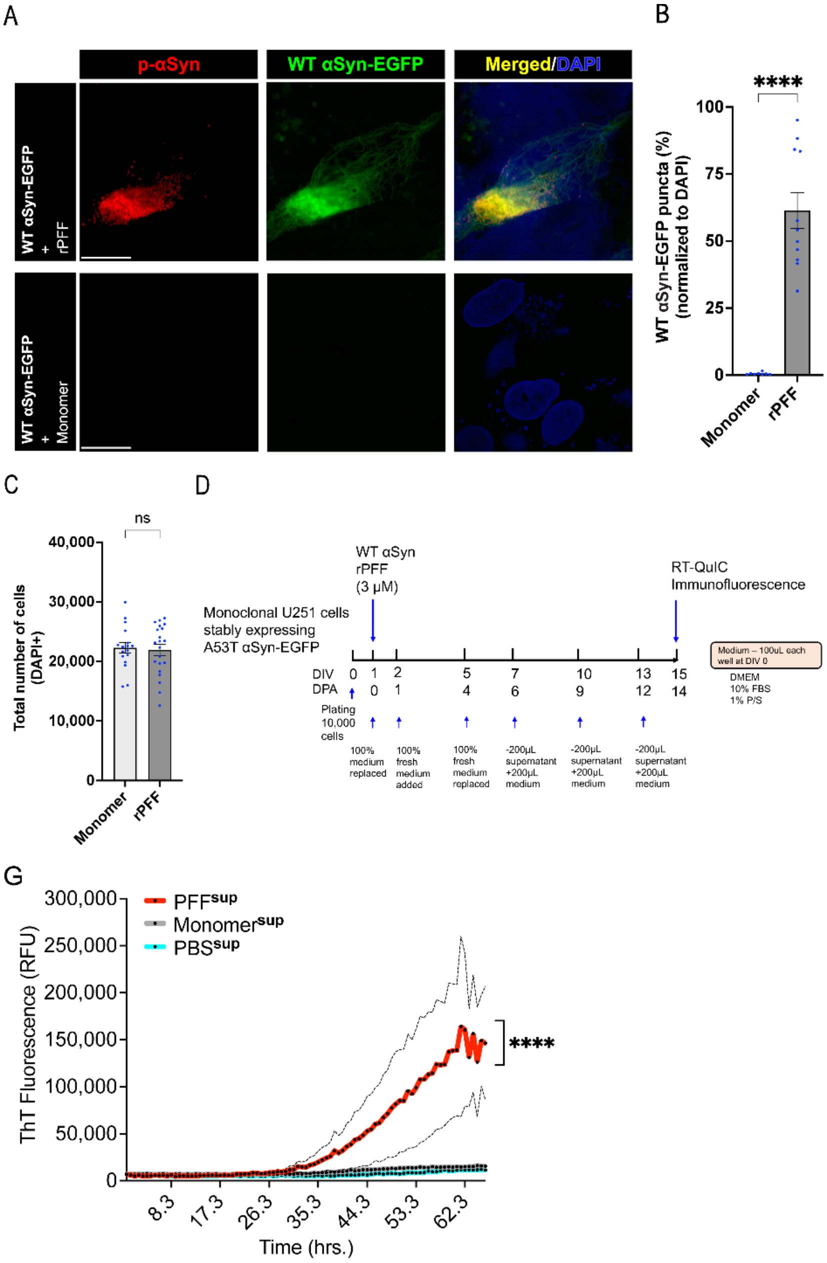

A
p-αSyn
WT αSyn-EGFP
Merged/DAPI
WT αSyn-EGFP + rPFF
WT αSyn-EGFP + Monomer
B
****
WT αSyn-EGFP puncta (%) (normalized to DAPI)
**
p-αSyn puncta (%) (normalized to DAPI)
ns
Total number of cells (DAPI+)
Monomer
rPFF
C
ns
Total number of cells (DAPI+)
D
WT αSyn rPFF (3 μM)
RT-QuIC Immunofluorescence
Monoclonal U251 cells stably expressing A53T αSyn-EGFP
DIV
DPA
Plating 10,000 cells
100% medium replaced
100% fresh medium added
100% fresh medium replaced
-200μL supernatant +200μL medium
Medium – 100uL each well at DIV 0
DMEM
10% FBS
1% P/S
E
****
A53T αSyn-EGFP puncta (%) (normalized to DAPI)
F
*
Total number of cells (DAPI+)
G
PFFsup
Monomersup
PBSsup
ThT Fluorescence (RFU)
****
Time (hrs.)

Fig. S4 rPFF induces intracellular aggregation of αSyn in U251 cells that release proteopathic αSyn aggregates.

(**A**) Representative confocal images showing pathological detergent-insoluble WT αSyn aggregates (green) that are phosphorylated (red). Cells were analyzed at DIV 5 (DPA 4) after in situ extraction of soluble proteins. Scale bars: 5 μm.

(**B**) Quantification of cells with detergent-insoluble WT αSyn-EGFP puncta (B, left), p-αSyn positive puncta (middle), and total number of cells (B, right) in each group at DPA 4. Data are presented as mean ± SEM, and each dot corresponds to a well where 15,000 to 25,000 cells were analyzed; N=3 per group from 2-3 independent experiments.

(**C**) Quantification of total number of A53T αSyn-EGFP expressing cells in each group at DPA 4. Data are presented as mean ± SEM, and each dot corresponds to a replicate where 15,000 to 25,000 cells were analyzed; N=3-4 per group from 4 independent experiments.

(**D**) Experimental design showing the timeline of αSyn-rPFF application on A53T αSyn-EGFP U251 cells with several medium changes.

(**E, F**) Quantification of A53T αSyn-EGFP puncta (E) and total number of cells in each group at DPA14 (F). Data are presented as mean ± SEM, and each dot corresponds to data analyzed from 10,000 to 15,000 cells in a well; N=9-10 per group.

(**G**) Detection of αSyn seeding activity from the supernatant of rPFF-treated A53T αSyn-EGFP U251 cells by RT-QuIC, but not from the supernatant of αSyn monomer or PBS treated cells at DPA 14. Data are presented as mean ± SEM (dashed line), and each dot represents data from N=2-5 replicates per group.

Statistical analysis was performed by ordinary two-way ANOVA with Tukey's multiple comparison test (G) or unpaired two-tailed Mann Whitney test or unpaired two-tailed student's t-test, and Welch's correction was applied when variances were found to be significant. A p-value <0.05 was considered significant. Significant differences are indicated by *$p<0.05$, **$p<0.01$, ***$p<0.001$, ****$p<0.0001$. Statistical power is > 0.90 between groups with p-value < 0.05.

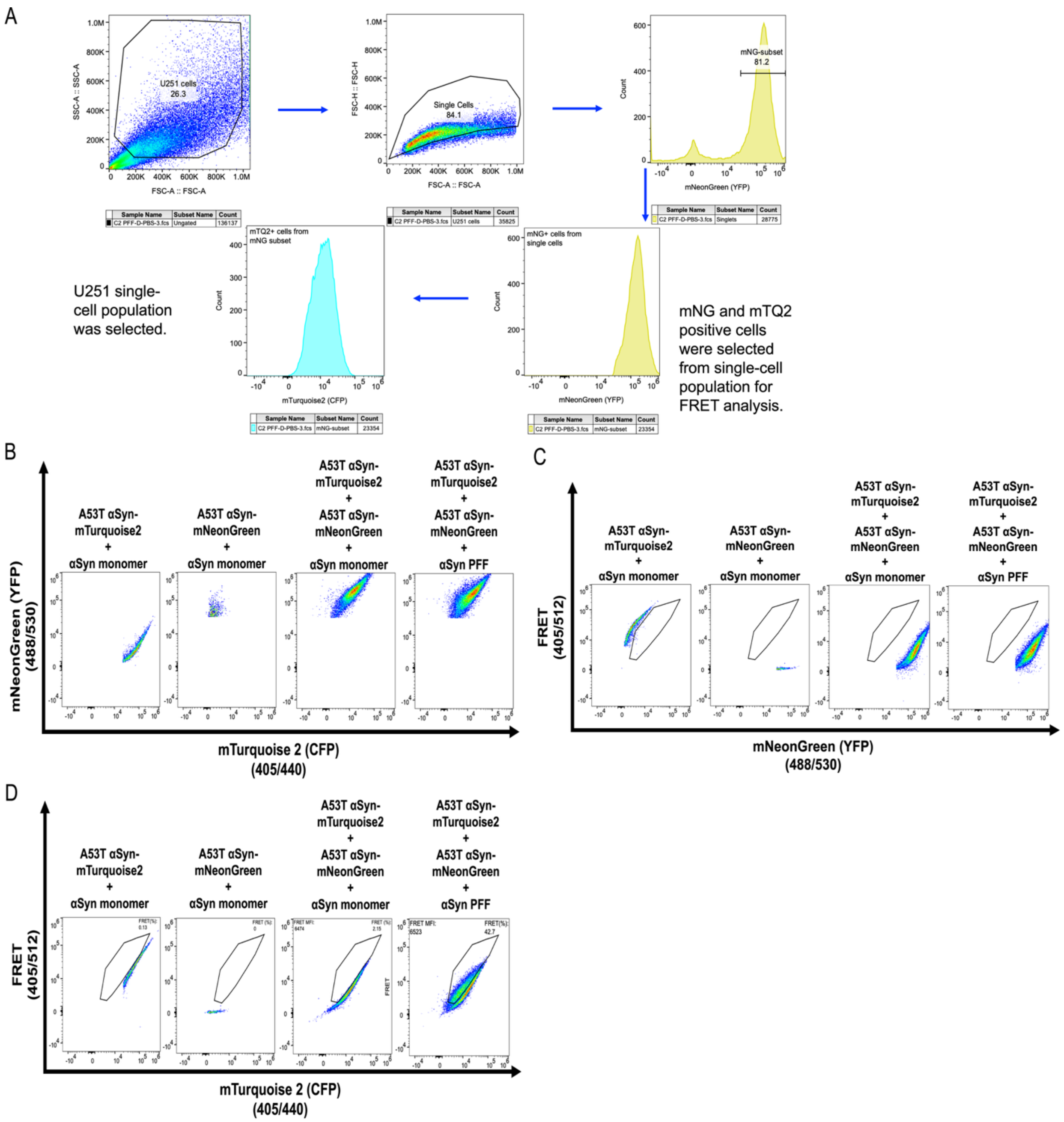

Fig. S5 FRET gating strategy to quantify percent FRET positive cells using FRET-Flow cytometry.

(**A**) Representative plots with gating to detect single U251 biosensor cells that express both αSyn-mTurquoise2 and αSyn mNeonGreen using FRET-flow cytometry.

(**B, C, D**) Representative plots with appropriately designed gates to detect FRET with the indicated combination of lasers (excitation/emission) showing the cellular expression profile of mTurquoise2 or mNeonGreen or FRET with either αSyn monomer or rPFF treatment.

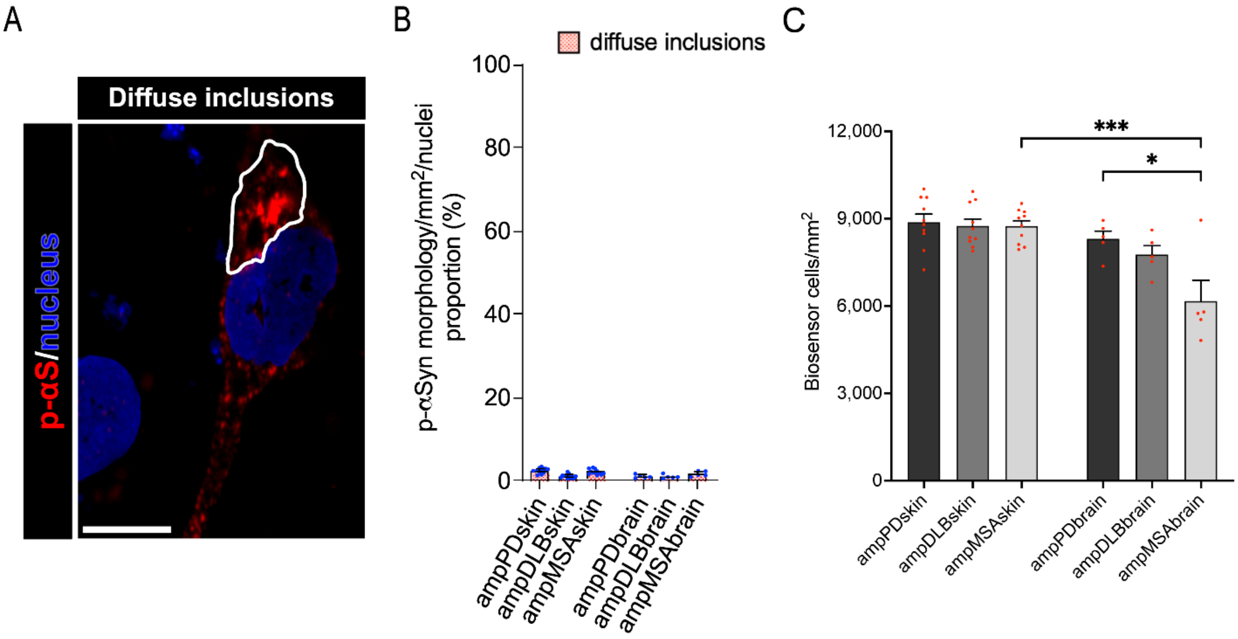

Fig. S6 Diffuse inclusion pathology induced by patient skin- and brain-amplified αSyn strains in biosensor cells.

(A) Representative confocal images showing diffuse inclusion morphology positive for p-αSyn in biosensor cells. Scale bar: 10 μm.

(**B, C**) Quantification of proportion of diffuse inclusion morphology (B) and total biosensor cells (C) at DPA 4. Data are presented as mean ± SEM, and each dot corresponds to data analyzed from 6,000 to 10,000 biosensor cells in a well from 2-3 independent experiments; N=10-12 from the skin-amplified strains of 2 cases and N=5 from the brain-amplified strains of 1 case.

Statistical analysis was performed by ordinary two-way (B) or one-way (C) ANOVA with Tukey's multiple comparison test. A p value < 0.05 was considered significant. Significant differences are indicated by *$p<0.05$, **$p<0.01$, ***$p<0.001$. Statistical power is > 0.90 between groups with p-value < 0.05.

A

Sarkosyl insoluble fraction

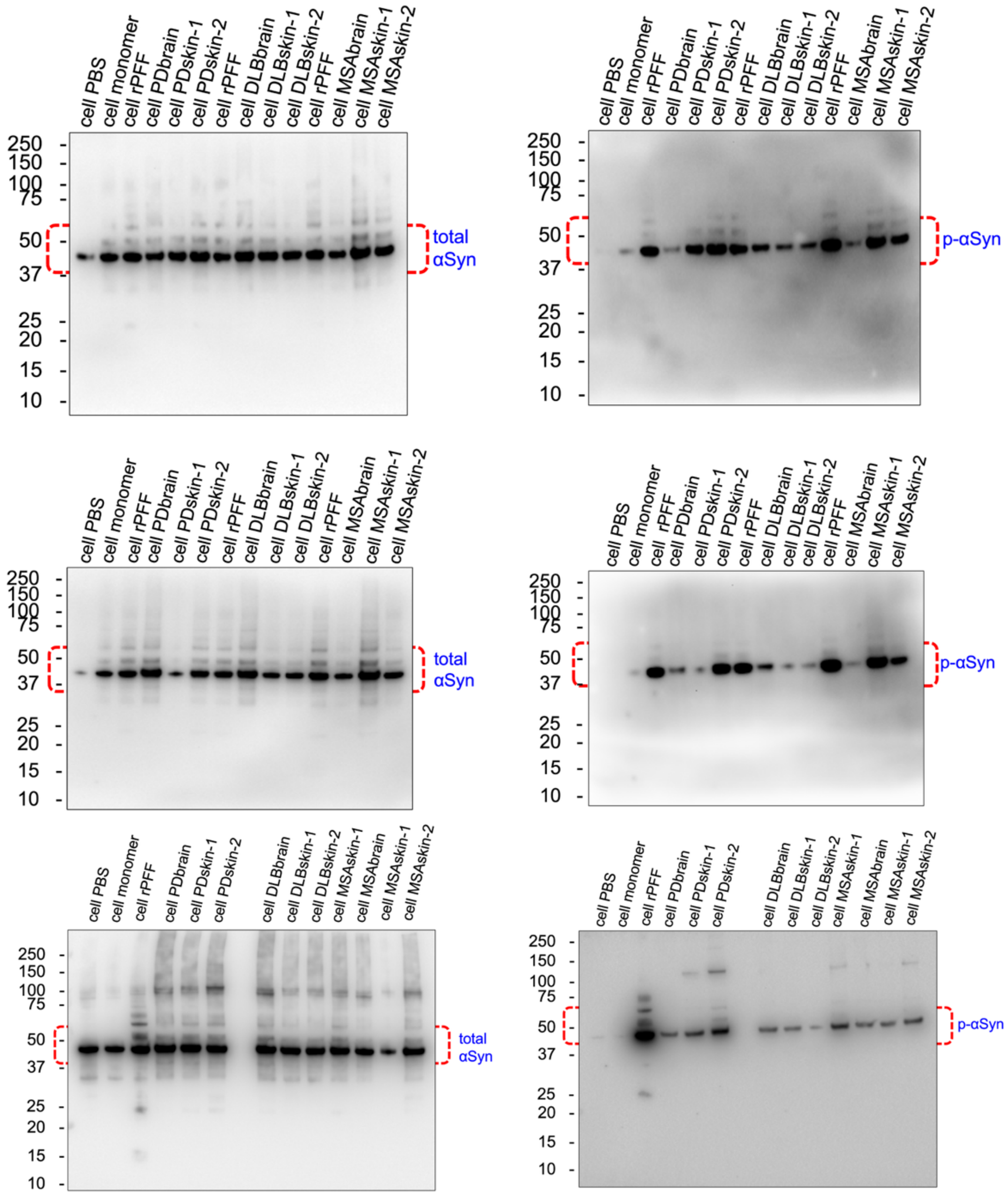

B

Sarkosyl soluble fraction

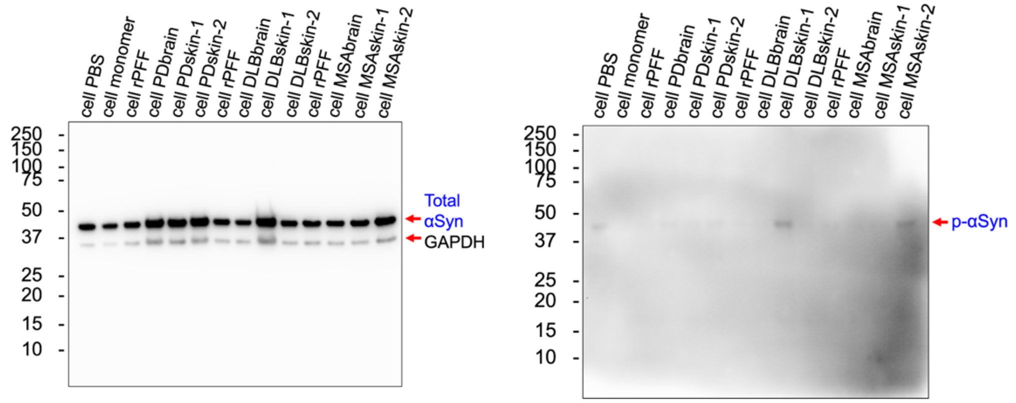

Fig. S7 Detection of total αSyn and p-αSyn in sarkosyl insoluble and sarkosyl soluble fractions from biosensor cells.

(**A, B)** Uncropped Western blots showing total αSyn (left) and pathological p-αSyn (right) detected in sarkosyl insoluble (A) and sarkosyl soluble (B) fractions from U251 biosensor cells seeded with patient skin- or brain-amplified αSyn strains. The red dashed boxes indicate the area on the blot used for densitometric analysis.
